# Supplementary material for: Potential efficacy of caffeine ingestion on balance and mobility in patients with multiple sclerosis: Preliminary evidence from a single-arm pilot clinical trial
Source: PLoS One. 2024 Feb 13;19(2):e0297235. doi: 10.1371/journal.pone.0297235 (PMC10863863; doi:10.1371/journal.pone.0297235)
Supplement: S1 File — The supplementary material file includes Table S1: The CONSORT checklist of information to include when reporting a pilot trial, Table S2: The 12-item Multiple Sclerosis Walking Scale (MSWS-12), Table S3: The Berg Balance Scale (BBS), Figure S1: The Timed Up-and-Go (TUG), Table S4: The Multiple Sclerosis Impact Scale (MSIS-29), Table S5: The Patient’s Global Impression of Changes (PGIC), and Figure S2: The impact of sex on each criterion during the study period. Supplementary Materials (containing the CONSORT checklist): Supplementary Materials (DOCX) [file pone.0297235.s001.docx]

**Supplementary Materials**

| **Table of Contents** | | **Page No.** |
| --- | --- | --- |
| **Table S1** | **The CONSORT checklist of information to include when reporting a pilot trial** | [**1-2**](#_Table_S1._The) |
| **Table S2** | **The 12-item Multiple Sclerosis Walking Scale (MSWS-12)** | [**3**](#_Table_S2._The) |
| **Table S3** | **The Berg Balance Scale (BBS)** | [**4-6**](#_Table_S3._The) |
| **Figure S1** | **The Timed Up-and-Go (TUG)** | [**7**](#_Figure_S1._The) |
| **Table S4** | **The Multiple Sclerosis Impact Scale (MSIS-29)** | [**8**](#_Table_S4._The) |
| **Table S5** | **The Patient’s Global Impression of Changes (PGIC)** | [**9**](#_Table_S5._The) |
| **Figure S2** | **The impact of sex on each criterion during the study period** | [**10**](#_Figure_S2._The) |
| **Table S6** | **The Spearman correlation coefficients for the total scores at different time points** | [**11-13**](#_Table_S6._The) |
| **Table S7** | **Confidence intervals of Spearman's rank correlation coefficients at different time points** | [**14-15**](#_Table_S7._Confidence) |

# **Table S1. The CONSORT checklist of information to include when reporting a pilot trial**


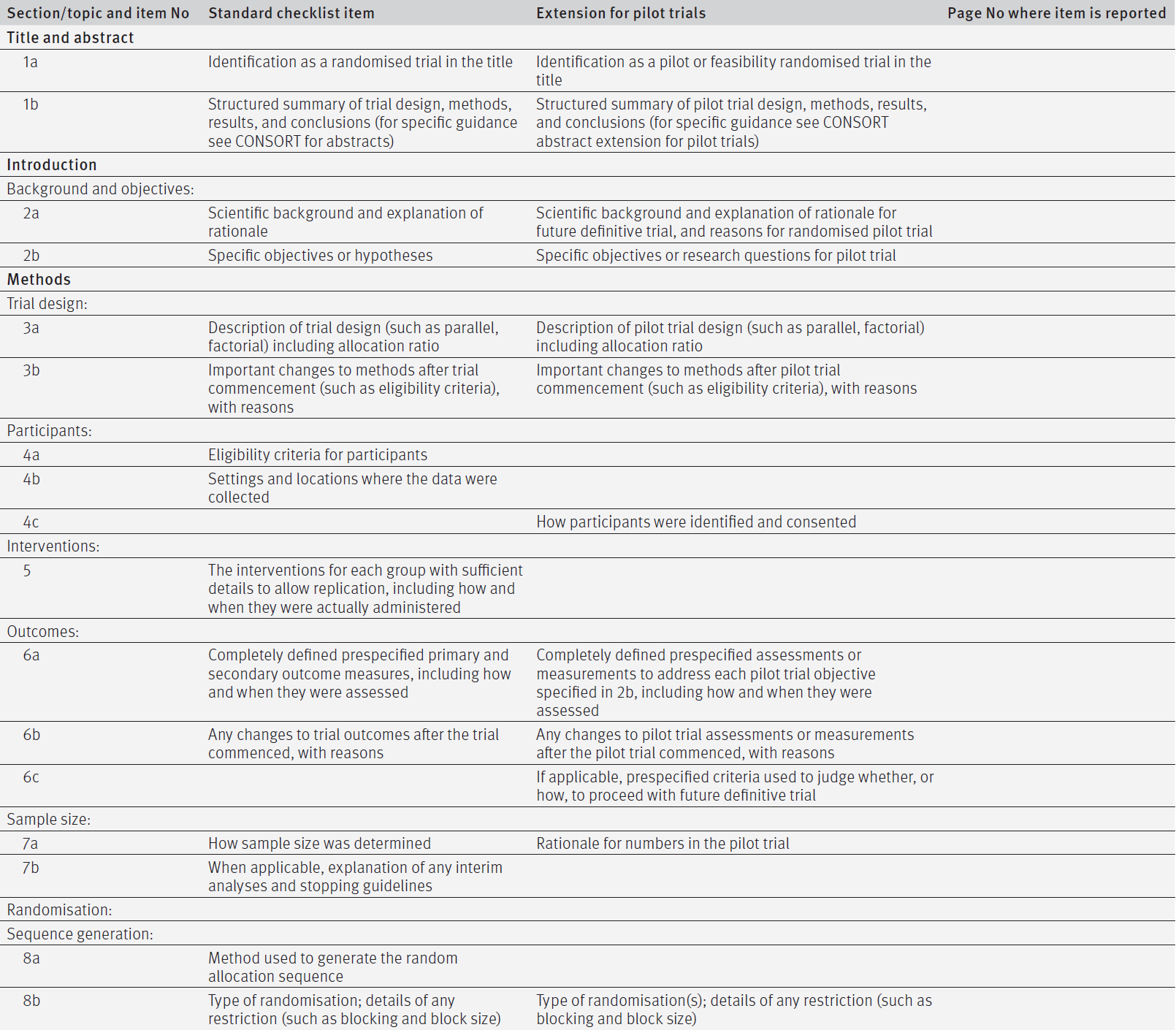

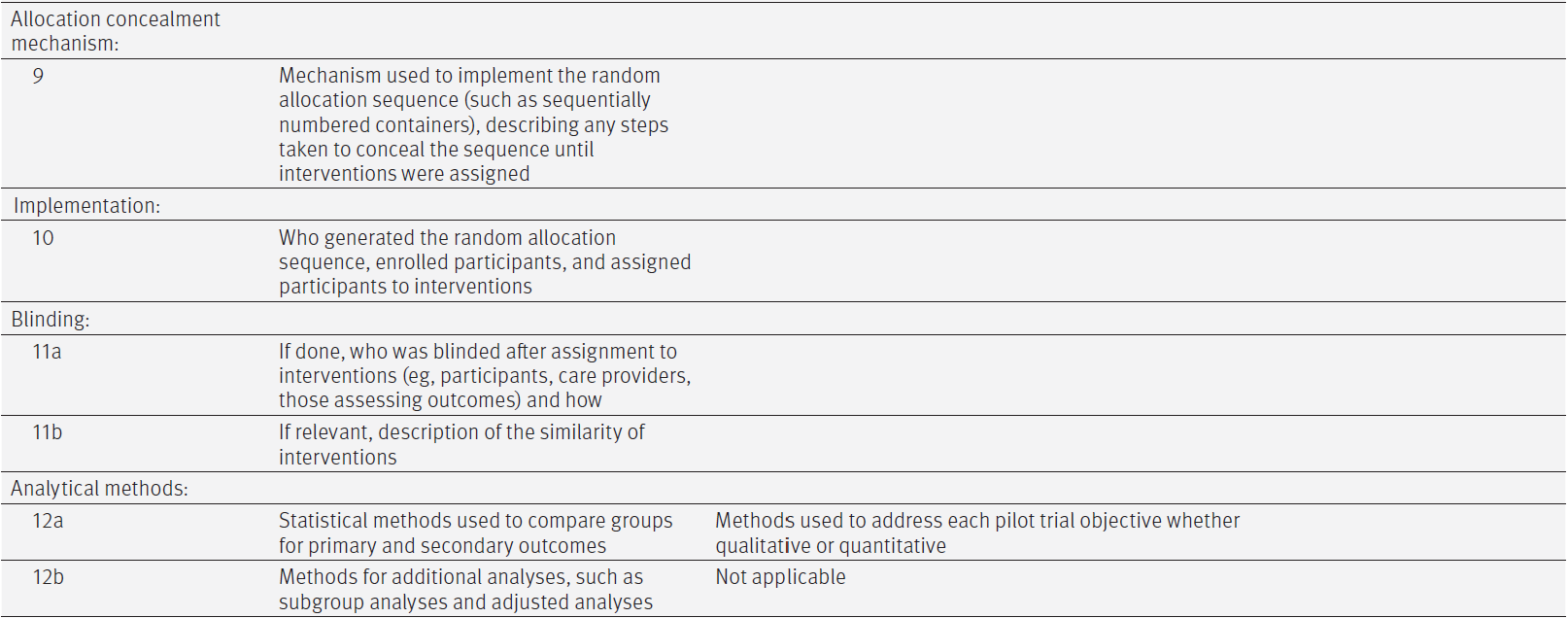


Page 9

Page 5

Page 6

Page 4

Page 4

Page 4 and Figure 1, Allocation: NA

Page 2

Page 2, 3

Page 1

Page 1

Page 11

Page 11

Page 9

NA

Page 9

NA

NA

NA

NA

NA

NA

Page 7

Pages 7

Available online at [CONSORT 2010 statement: extension to randomised pilot and feasibility trials | The BMJ](https://www.bmj.com/content/355/bmj.i5239)

Page 17-19

Page 19,20

Page 19,20

Page 22

Page 22

Page 6 (March 9, 2017-January 2, 2018)

NA

Page 22

Page 19-21

NA

Page 12

Page 13-16

Page 13,14,15,16

Page 13, Figure 2

Page 12, Table 1

NA

Page 12

Page 12, Figure 2


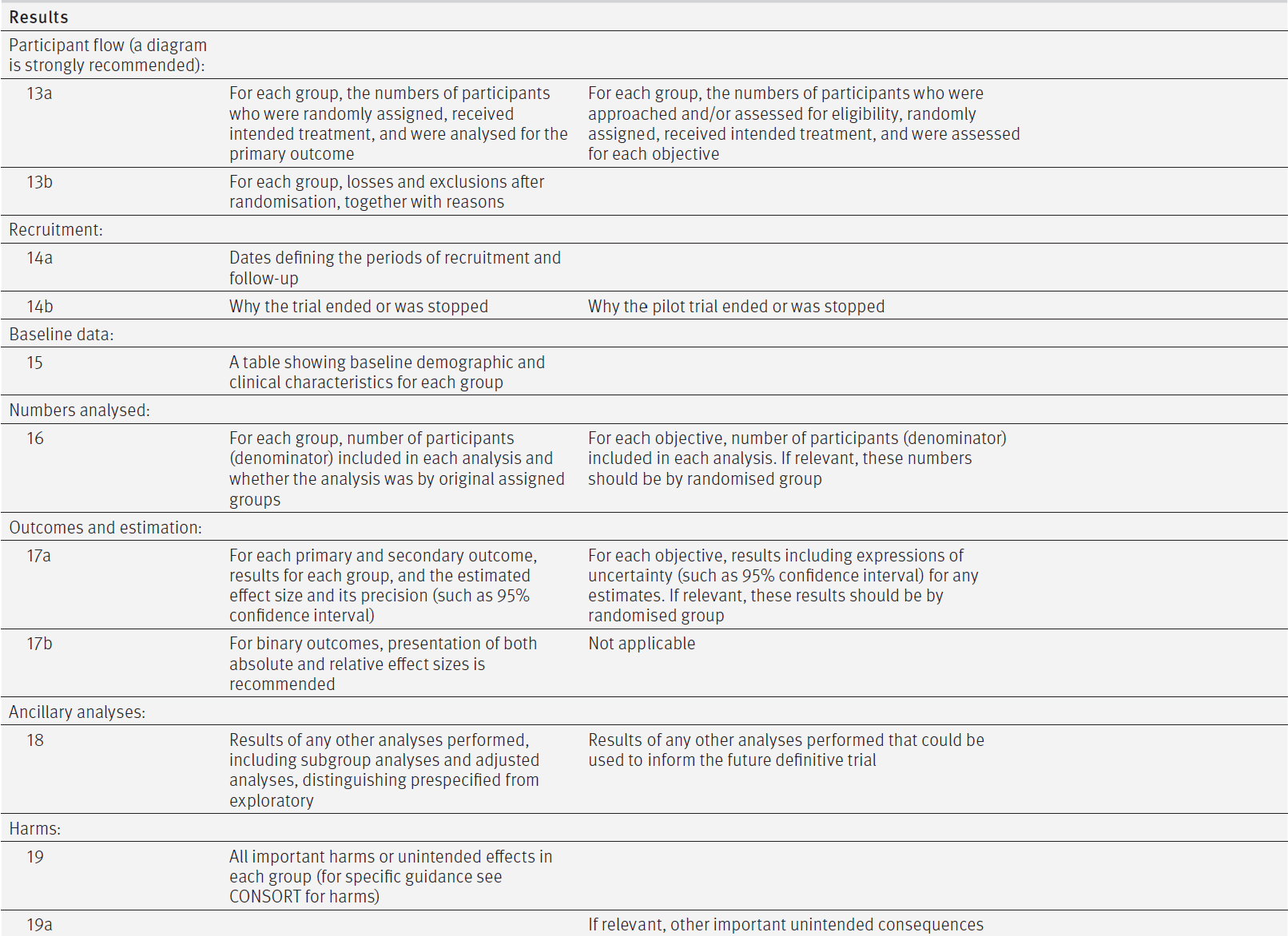

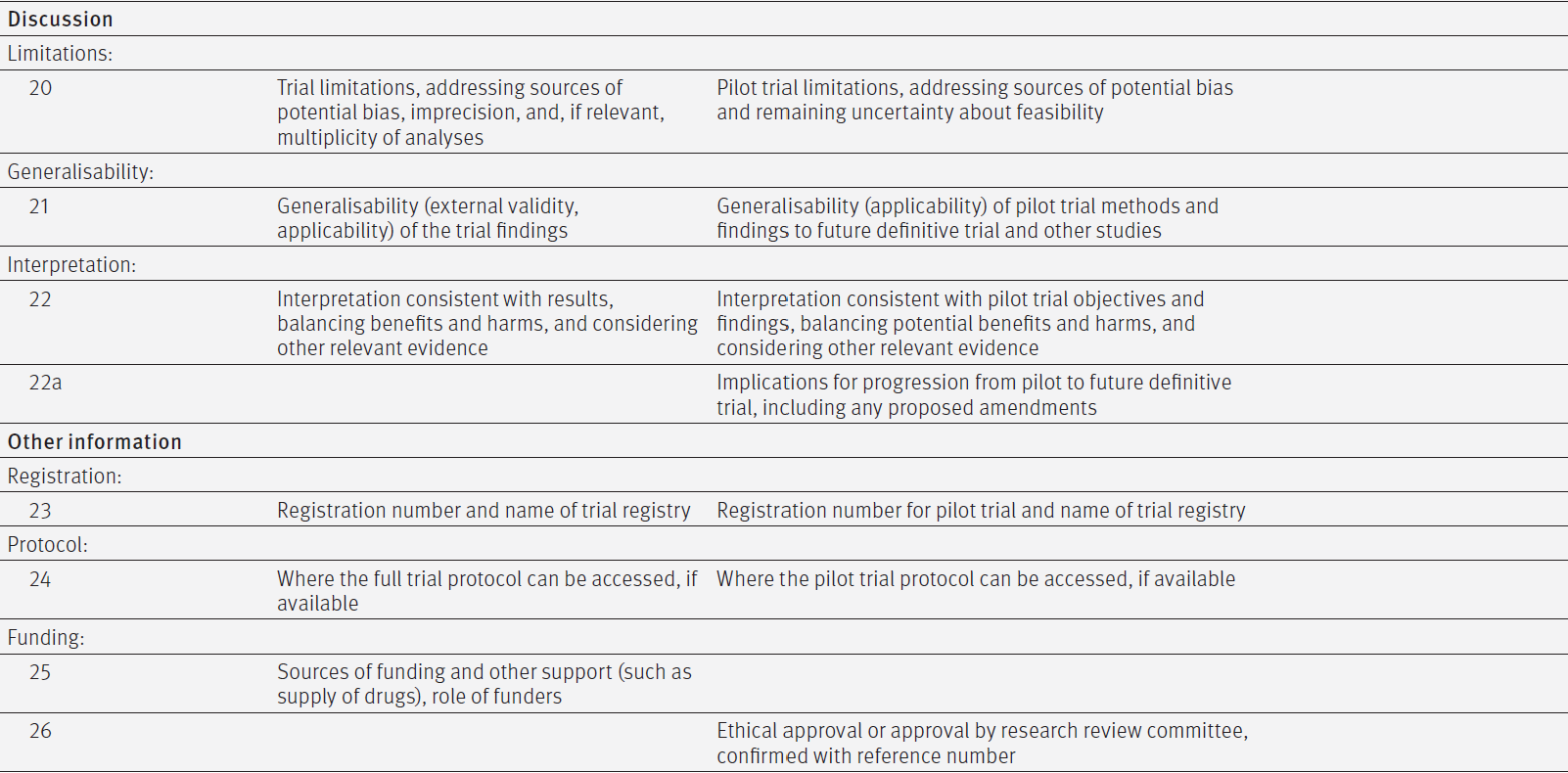


# **Table S2. The 12-item Multiple Sclerosis Walking Scale (MSWS-12)**

| **In the past two weeks,**  **how much has your MS . . .** | **Not at all** | **A little** | **Moderately** | **Quite a lot** | **Extremely** |
| --- | --- | --- | --- | --- | --- |
| 1. Limited your ability to walk? | 1 | 2 | 3 | 4 | 5 |
| 2. Limited your ability to run? | 1 | 2 | 3 | 4 | 5 |
| 3. Limited your ability to climb up and down stairs? | 1 | 2 | 3 | 4 | 5 |
| 4. Made standing when doing things more difficult? | 1 | 2 | 3 | 4 | 5 |
| 5. Limited your balance when standing or walking? | 1 | 2 | 3 | 4 | 5 |
| 6. Limited how far you can walk? | 1 | 2 | 3 | 4 | 5 |
| 7. Increased the effort needed for you to walk? | 1 | 2 | 3 | 4 | 5 |
| 8. Made it necessary for you to use support when walking indoors (e.g., holding on to furniture, using a stick, etc.)? | 1 | 2 | 3 | 4 | 5 |
| 9. Made it necessary for you to use support when  walking outdoors (e.g., using a stick, a frame, etc.)? | 1 | 2 | 3 | 4 | 5 |
| 10. Slowed down your walking? | 1 | 2 | 3 | 4 | 5 |
| 11. Affected how smoothly you walk? | 1 | 2 | 3 | 4 | 5 |
| 12. Made you concentrate on your walking? | 1 | 2 | 3 | 4 | 5 |

Available online at: [msws-eng.pdf (sralab.org)](https://www.sralab.org/sites/default/files/2017-07/msws-eng.pdf)

# **Table S3. The Berg Balance Scale (BBS)**

| **Item** | **Instruction** | **Scoring** |
| --- | --- | --- |
| 1. **Sitting to standing** | Please stand up. Try not to use your hand for support. | - 4: able to stand without using hands and stabilize independently - 3: able to stand independently using hands - 2: able to stand using hands after several tries - 1: needs minimal aid to stand or stabilize - 0: needs moderate or maximal assistance to stand |
| 1. **Standing unsupported** | Please stand for two minutes without holding on. | - 4: able to stand safely for 2 minutes - 3: able to stand for 2 minutes with supervision - 2: able to stand 30 seconds unsupported - 1: needs several tries to stand 30 seconds unsupported - 0: unable to stand for 30 seconds unsupported |
| **3. Sitting with back unsupported but feet supported on the floor or a stool** | Please sit with your arms folded for 2 minutes. | - 4: able to sit safely and securely for 2 minutes - 3: able to sit for 2 minutes under supervision - 2: able to sit for 30 seconds - 1: able to sit for 10 seconds - 0: unable to sit without support for 10 seconds |
| **4. Standing to sitting** | Please sit down. | - 4: sits safely with minimal use of hands - 3: controls descent by using hands - 2: uses the back of legs against a chair to control descent - 1: sits independently but has uncontrolled descent - 0: needs assist to sit |
| **5. Transfers** | Arrange chair(s) for pivot transfer. Ask the subject to transfer one way toward a seat with armrests and one way toward a seat without armrests. You may use two chairs (one with and one without armrests) or a bed and a chair. | - 4: able to transfer safely with minor use of hands - 3: able to transfer safely definite need of hands - 2: able to transfer with verbal cueing and/or supervision - 1: needs one person to assist - 0: needs two people to assist or supervise to be safe |
| **6. Standing unsupported with eyes closed** | Please close your eyes and stand still for 10 seconds. | - 4: able to stand for 10 seconds safely - 3: able to stand for 10 seconds with supervision - 2: able to stand 3 seconds - 1: unable to keep eyes closed 3 seconds but stays safely - 0: needs help to keep from falling |
| **7. Standing unsupported with feet together** | Place your feet together and stand without holding on. | - 4: able to place feet together independently and stand 1 minute safely - 3: able to place feet together independently and stand for 1 minute with supervision - 2: able to place feet together independently but unable to hold for 30 seconds - 1: needs help to attain position but able to stand 15 seconds feet together - 0: needs help to attain position and is unable to hold for 15 seconds |
| **8. Reaching forward with outstretched arms while standing** | Lift arm to 90 degrees. Stretch out your fingers and reach forward as far as you can. (The examiner places a ruler at the end of the fingertips when the arm is at 90 degrees. Fingers should not touch the ruler while reaching forward. The recorded measure is the distance forward that the fingers reach while the subject is in the most forward lean position. When possible, ask the subject to use both arms when reaching to avoid rotation of the trunk.) | - 4: can reach forward confidently 25 cm (10 inches) - 3: can reach forward 12 cm (5 inches) - 2: can reach forward 5 cm (2 inches) - 1: reaches forward but needs supervision - 0: loses balance while trying/requires external support |
| **9. Pick up an object from the floor from a standing position** | Pick up the shoe/slipper, which is placed in front of your feet. | - 4: able to pick up slipper safely and easily - 3: able to pick up slippers but needs supervision - 2: unable to pick up but reaches 2-5 cm (1-2 inches) from slipper and keeps balance independently - 1: unable to pick up and needs supervision while trying - 0: unable to try/needs assistance to keep from losing balance or falling |
| **10. Turing to look behind overt left and right shoulders while standing** | Turn to look directly behind you over toward the left shoulder. Repeat to the right. The examiner may pick an object to look at directly behind the subject to encourage a better twist turn. | - 4: looks behind from both sides and weight shifts well - 3: looks behind one side only the other side shows less weight shift - 2: turns sideways only but maintains balance - 1: needs supervision when turning - 0: needs assistance to keep from losing balance or falling |
| **11. Turn 360 degrees** | Turn completely around in a full circle. Pause. Then turn a full circle in the other direction. | - 4: able to turn 360 degrees safely in 4 seconds or less - 3: able to turn 360 degrees safely on one side in only 4 seconds or less - 2: able to turn 360 degrees safely but slowly - 1: needs close supervision or verbal cueing - 0: needs assistance while turning |
| **12. Place alternate foot on a step or stool while standing unsupported** | Place each foot alternately on the step/stool. Continue until each foot has touched the step/stool four times | - 4: able to stand independently and safely and complete 8 steps in 20 seconds - 3: able to stand independently and complete 8 steps in > 20 seconds - 2: able to complete 4 steps without aid with supervision - 1: able to complete > 2 steps needs minimal assist - 0: needs assistance to keep from falling/unable to try |
| **13. Standing unsupported on one foot in front** | Place one foot directly in front of the other. If you feel that you cannot place your foot directly in front, try to step far enough ahead that the heel of your forward foot is ahead of the toes of the other foot. (To score 3 points, the length of the step should exceed the length of the other foot and the width of the stance should approximate the subject’s normal stride width.) | - 4: able to place foot tandem independently and hold for 30 seconds - 3: able to place foot ahead independently and hold for 30 seconds - 2: able to take small steps independently and hold for 30 seconds - 1: needs help to step but can hold for 15 seconds - 0 loses balance while stepping or standing |
| **14. Standing on one leg** | Stand on one leg as long as you can without holding on. | - 4: able to lift leg independently and hold > 10 seconds - 3: able to lift leg independently and hold for 5-10 seconds - 2: able to lift leg independently and hold ≥ 3 seconds - 1: tries to lift leg unable to hold 3 seconds but remains standing independently - 0: unable to try or needs assistance to prevent fall |

Available at: [brandeis.edu/roybal/docs/Berg-Balance-Scale_Website.pdf](https://www.brandeis.edu/roybal/docs/Berg-Balance-Scale_Website.pdf)

# **Figure S1. The Timed Up-and-Go (TUG)**


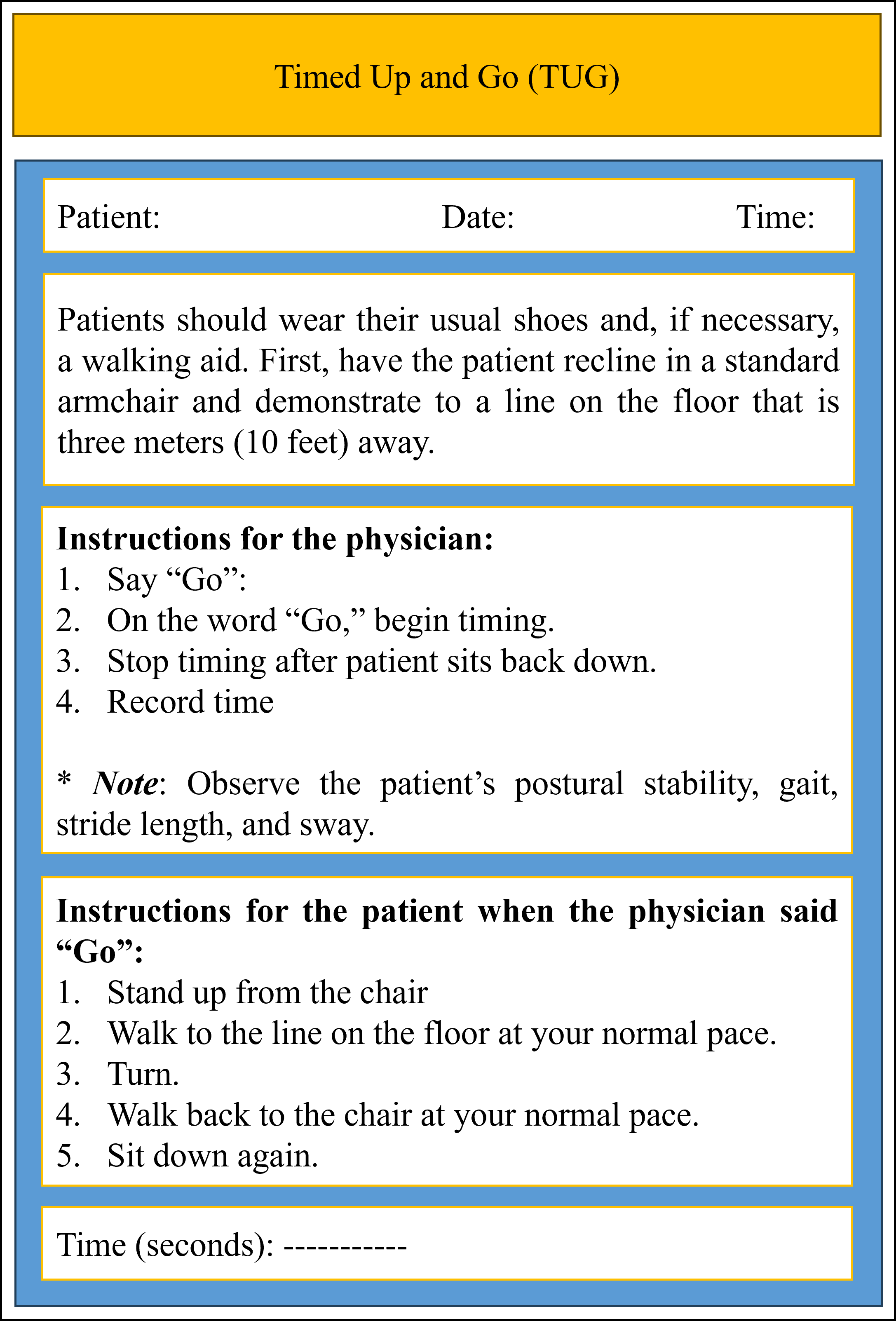


Figure S1. The figure is similar but not identical to the original image from [TUG_test-print.pdf (cdc.gov)](https://www.cdc.gov/steadi/pdf/TUG_test-print.pdf) and is therefore for illustrative purposes only.

# **Table S4. The Multiple Sclerosis Impact Scale (MSIS-29)**

|  | **Not at all** | **A little** | **Moderately** | **Quite a bit** | **Extremely** |
| --- | --- | --- | --- | --- | --- |
| **In the past two weeks, how much has your MS limited your ability to...** | | | | | |
| 1. Do physically demanding tasks? | 1 | 2 | 3 | 4 | 5 |
| 2. Grip things tightly (e.g., turning on taps)? | 1 | 2 | 3 | 4 | 5 |
| 3. Carry things? | 1 | 2 | 3 | 4 | 5 |
| **In the past two weeks, how much have you been bothered by...** | | | | | |
| 4. Problems with your balance? | 1 | 2 | 3 | 4 | 5 |
| 5. Difficulties moving about indoors? | 1 | 2 | 3 | 4 | 5 |
| 6. Being clumsy? | 1 | 2 | 3 | 4 | 5 |
| 7. Stiffness? | 1 | 2 | 3 | 4 | 5 |
| 8. Heavy arms and/or legs? | 1 | 2 | 3 | 4 | 5 |
| 9. Tremor of your arms or legs? | 1 | 2 | 3 | 4 | 5 |
| 10. Spasms in your limbs? | 1 | 2 | 3 | 4 | 5 |
| 11. Your body not doing what you want it to do? | 1 | 2 | 3 | 4 | 5 |
| 12. Having to depend on others to do things for you? | 1 | 2 | 3 | 4 | 5 |
| 13. Limitations in your social and leisure activities at home? | 1 | 2 | 3 | 4 | 5 |
| 14. Being stuck at home more than you would like to be? | 1 | 2 | 3 | 4 | 5 |
| 15. Difficulties using your hands in everyday tasks? | 1 | 2 | 3 | 4 | 5 |
| 16. Having to cut down the amount of time you spent on work or other daily activities? | 1 | 2 | 3 | 4 | 5 |
| 17. Problems using transport (e.g., car, bus, train, taxi, etc.)? | 1 | 2 | 3 | 4 | 5 |
| 18. Taking longer to do things? | 1 | 2 | 3 | 4 | 5 |
| 19. Difficulty doing things spontaneously (e.g. going out on the spur of the moment)? | 1 | 2 | 3 | 4 | 5 |
| 20. Needing to go to the toilet urgently? | 1 | 2 | 3 | 4 | 5 |
| 21. Feeling unwell? | 1 | 2 | 3 | 4 | 5 |
| 22. Problems sleeping? | 1 | 2 | 3 | 4 | 5 |
| 23. Feeling mentally fatigued? | 1 | 2 | 3 | 4 | 5 |
| 24. Worries related to your MS? | 1 | 2 | 3 | 4 | 5 |
| 25. Feeling anxious or tense? | 1 | 2 | 3 | 4 | 5 |
| 26. Feeling irritable, impatient, or short-tempered? | 1 | 2 | 3 | 4 | 5 |
| 27. Problems concentrating? | 1 | 2 | 3 | 4 | 5 |
| 28 Lack of confidence? | 1 | 2 | 3 | 4 | 5 |
| 29. Feeling depressed? | 1 | 2 | 3 | 4 | 5 |

Available at: [Microsoft Word - MSIS - 29.doc (mstrust.org.uk)](https://mstrust.org.uk/sites/default/files/MSIS-29.pdf)

# **Table S5. The Patient’s Global Impression of Changes (PGIC)**

| **Since the beginning of the treatment at this clinic, how would you describe the change (if any) in your activity limitations, symptoms, emotions, and overall quality of your life attributed to your condition?** | |
| --- | --- |
| No change (or the condition has got worse) | **1** |
| Almost the same, hardly any change at all | **2** |
| A little better, but no noticeable change | **3** |
| Somewhat better, but the change has not made any real difference | **4** |
| Moderately better, and a slight but noticeable change | **5** |
| Better, and a definite improvement that has made a real and worthwhile difference | **6** |
| A great deal better, and a considerable improvement that has made all the difference | **7** |

Available at: [Website (mapi-trust.org)](https://eprovide.mapi-trust.org/instruments/patient-global-impressions-scale-change-improvement-severity)

# **Figure S2. The impact of sex on each criterion during the study period**

**Figure S2.** The impact of sex on each criterion during the study period. Abbreviations: MSWS-12: 12-item Multiple Sclerosis Walking Scale, BBS: Berg Balance Scale, TUG: Timed Up-and-Go, MSIS-29: Multiple Sclerosis Impact Scale, PGIC: Patients’ Global Impression of Change.


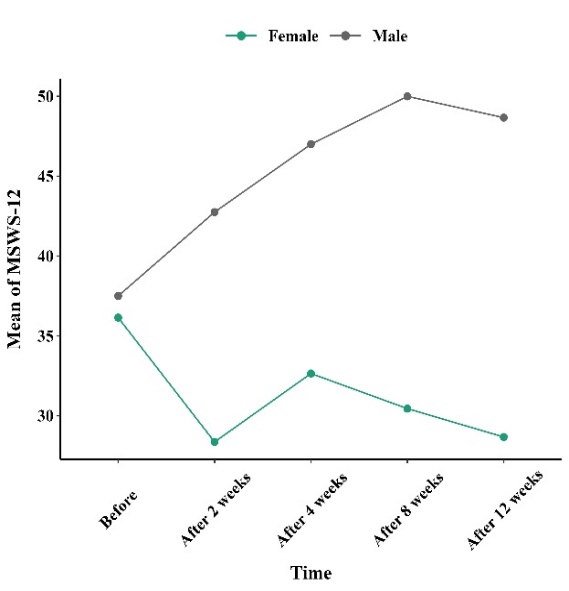

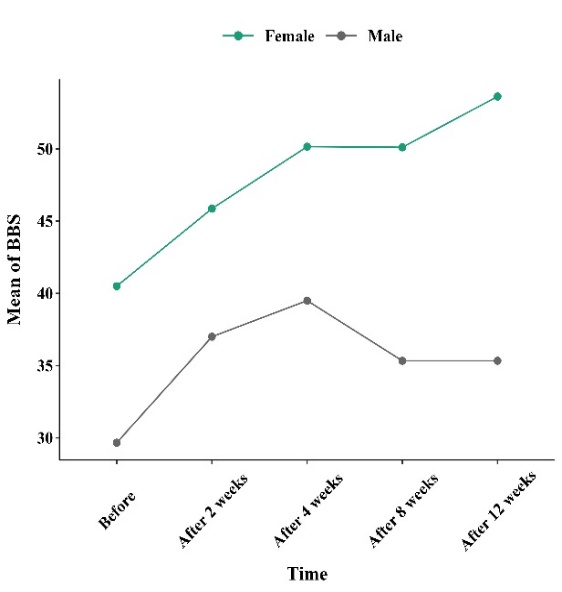

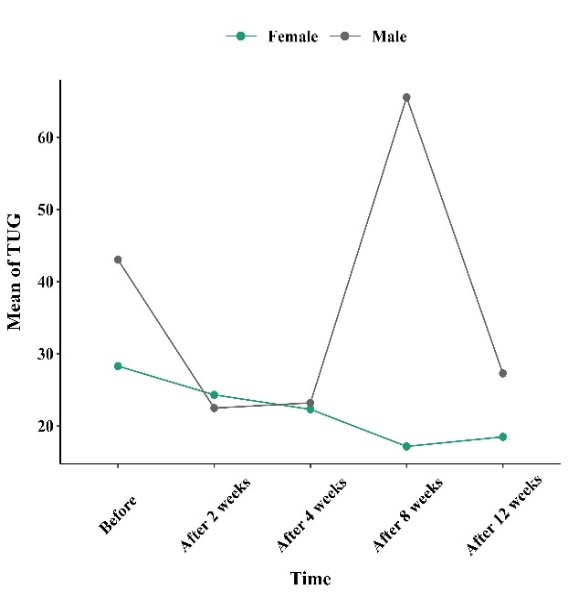

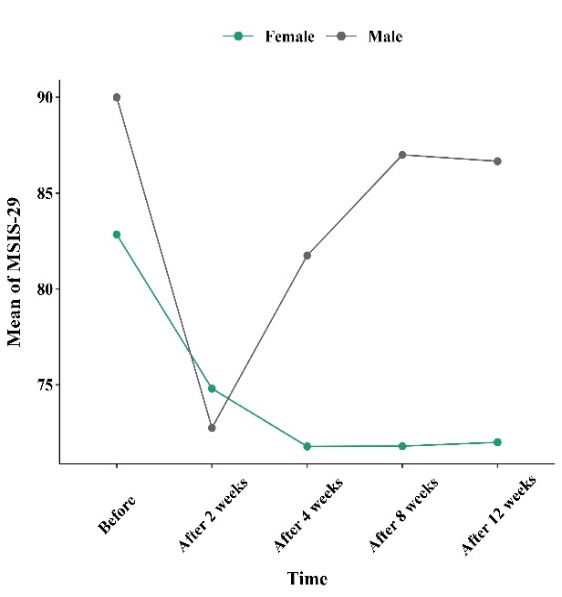

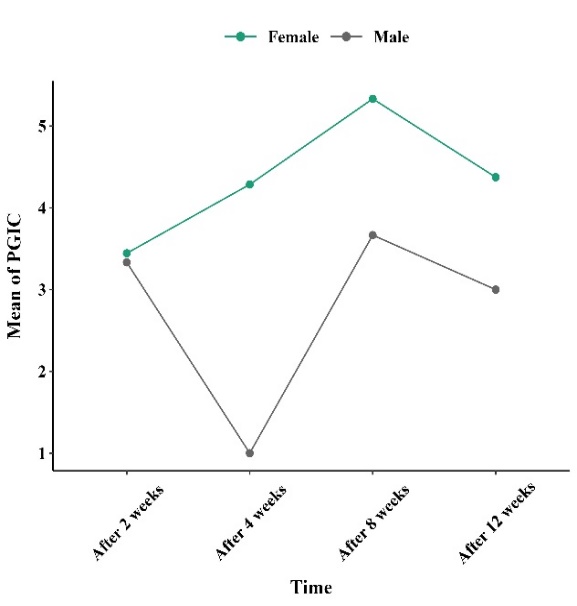


# **Table S6. The Spearman correlation coefficients for the total scores at different time points**

| **Correlations** | | | | | | | | |
| --- | --- | --- | --- | --- | --- | --- | --- | --- |
| Time | | | | **BBS** | **TUG** | **PGIC** | **MSWS-12** | **MSIS** |
| Before | Spearman's rho | BBS | Correlation Coefficient | 1.000 | -.820^**^ | . | -.440^*^ | -.498^*^ |
|  |  |  | Sig. (2-tailed) | . | .000 | . | .022 | .011 |
|  |  |  | N | 27 | 25 | 0 | 27 | 25 |
|  |  | TUG | Correlation Coefficient | -.820^**^ | 1.000 | . | .642^**^ | .732^**^ |
|  |  |  | Sig. (2-tailed) | .000 | . | . | .000 | .000 |
|  |  |  | N | 25 | 26 | 0 | 26 | 24 |
|  |  | PGIC | Correlation Coefficient | . | . | . | . | . |
|  |  |  | Sig. (2-tailed) | . | . | . | . | . |
|  |  |  | N | 0 | 0 | 0 | 0 | 0 |
|  |  | MSWS-12 | Correlation Coefficient | -.440^*^ | .642^**^ | . | 1.000 | .822^**^ |
|  |  |  | Sig. (2-tailed) | .022 | .000 | . | . | .000 |
|  |  |  | N | 27 | 26 | 0 | 28 | 26 |
|  |  | MSIS-29 | Correlation Coefficient | -.498^*^ | .732^**^ | . | .822^**^ | 1.000 |
|  |  |  | Sig. (2-tailed) | .011 | .000 | . | .000 | . |
|  |  |  | N | 25 | 24 | 0 | 26 | 26 |
| After 2 weeks | Spearman's rho | BBS | Correlation Coefficient | 1.000 | -.944^**^ | .219 | -.748^**^ | -.265 |
|  |  |  | Sig. (2-tailed) | . | .000 | .368 | .000 | .287 |
|  |  |  | N | 22 | 16 | 19 | 18 | 18 |
|  |  | TUG | Correlation Coefficient | -.944^**^ | 1.000 | -.360 | .576^*^ | .241 |
|  |  |  | Sig. (2-tailed) | .000 | . | .155 | .016 | .352 |
|  |  |  | N | 16 | 17 | 17 | 17 | 17 |
|  |  | PGIC | Correlation Coefficient | .219 | -.360 | 1.000 | -.353 | -.620^**^ |
|  |  |  | Sig. (2-tailed) | .368 | .155 | . | .151 | .005 |
|  |  |  | N | 19 | 17 | 22 | 18 | 19 |
|  |  | MSWS-12 | Correlation Coefficient | -.748^**^ | .576^*^ | -.353 | 1.000 | .595^**^ |
|  |  |  | Sig. (2-tailed) | .000 | .016 | .151 | . | .007 |
|  |  |  | N | 18 | 17 | 18 | 19 | 19 |
|  |  | MSIS-29 | Correlation Coefficient | -.265 | .241 | -.620^**^ | .595^**^ | 1.000 |
|  |  |  | Sig. (2-tailed) | .287 | .352 | .005 | .007 | . |
|  |  |  | N | 18 | 17 | 19 | 19 | 20 |
| After 4 weeks | Spearman's rho | BBS | Correlation Coefficient | 1.000 | -.860^**^ | .448 | -.757^**^ | -.551^*^ |
|  |  |  | Sig. (2-tailed) | . | .000 | .082 | .001 | .022 |
|  |  |  | N | 17 | 13 | 16 | 16 | 17 |
|  |  | TUG | Correlation Coefficient | -.860^**^ | 1.000 | -.210 | .682^**^ | .744^**^ |
|  |  |  | Sig. (2-tailed) | .000 | . | .452 | .005 | .001 |
|  |  |  | N | 13 | 15 | 15 | 15 | 15 |
|  |  | PGIC | Correlation Coefficient | .448 | -.210 | 1.000 | -.416 | -.181 |
|  |  |  | Sig. (2-tailed) | .082 | .452 | . | .097 | .473 |
|  |  |  | N | 16 | 15 | 18 | 17 | 18 |
|  |  | MSWS-12 | Correlation Coefficient | -.757^**^ | .682^**^ | -.416 | 1.000 | .746^**^ |
|  |  |  | Sig. (2-tailed) | .001 | .005 | .097 | . | .000 |
|  |  |  | N | 16 | 15 | 17 | 19 | 18 |
|  |  | MSIS-29 | Correlation Coefficient | -.551^*^ | .744^**^ | -.181 | .746^**^ | 1.000 |
|  |  |  | Sig. (2-tailed) | .022 | .001 | .473 | .000 | . |
|  |  |  | N | 17 | 15 | 18 | 18 | 19 |
| After 8 weeks | Spearman's rho | BBS | Correlation Coefficient | 1.000 | -.757^*^ | .372 | -.826^**^ | -.557 |
|  |  |  | Sig. (2-tailed) | . | .049 | .324 | .002 | .060 |
|  |  |  | N | 12 | 7 | 9 | 11 | 12 |
|  |  | TUG | Correlation Coefficient | -.757^*^ | 1.000 | .057 | .778^*^ | .633 |
|  |  |  | Sig. (2-tailed) | .049 | . | .875 | .014 | .067 |
|  |  |  | N | 7 | 11 | 10 | 9 | 9 |
|  |  | PGIC | Correlation Coefficient | .372 | .057 | 1.000 | -.215 | -.005 |
|  |  |  | Sig. (2-tailed) | .324 | .875 | . | .525 | .989 |
|  |  |  | N | 9 | 10 | 12 | 11 | 11 |
|  |  | MSWS-12 | Correlation Coefficient | -.826^**^ | .778^*^ | -.215 | 1.000 | .691^**^ |
|  |  |  | Sig. (2-tailed) | .002 | .014 | .525 | . | .009 |
|  |  |  | N | 11 | 9 | 11 | 13 | 13 |
|  |  | MSIS-29 | Correlation Coefficient | -.557 | .633 | -.005 | .691^**^ | 1.000 |
|  |  |  | Sig. (2-tailed) | .060 | .067 | .989 | .009 | . |
|  |  |  | N | 12 | 9 | 11 | 13 | 14 |
| After 12 weeks | Spearman's rho | BBS | Correlation Coefficient | 1.000 | -.690^*^ | -.099 | -.478 | -.185 |
|  |  |  | Sig. (2-tailed) | . | .040 | .800 | .162 | .610 |
|  |  |  | N | 11 | 9 | 9 | 10 | 10 |
|  |  | TUG | Correlation Coefficient | -.690^*^ | 1.000 | .017 | .610 | .600 |
|  |  |  | Sig. (2-tailed) | .040 | . | .965 | .081 | .088 |
|  |  |  | N | 9 | 11 | 9 | 9 | 9 |
|  |  | PGIC | Correlation Coefficient | -.099 | .017 | 1.000 | -.512 | -.641 |
|  |  |  | Sig. (2-tailed) | .800 | .965 | . | .159 | .063 |
|  |  |  | N | 9 | 9 | 10 | 9 | 9 |
|  |  | MSWS-12 | Correlation Coefficient | -.478 | .610 | -.512 | 1.000 | .781^**^ |
|  |  |  | Sig. (2-tailed) | .162 | .081 | .159 | . | .005 |
|  |  |  | N | 10 | 9 | 9 | 12 | 11 |
|  |  | MSIS-29 | Correlation Coefficient | -.185 | .600 | -.641 | .781^**^ | 1.000 |
|  |  |  | Sig. (2-tailed) | .610 | .088 | .063 | .005 | . |
|  |  |  | N | 10 | 9 | 9 | 11 | 11 |
| **. Correlation is significant at the 0.01 level (2-tailed). | | | | | | | | |
| *. Correlation is significant at the 0.05 level (2-tailed). | | | | | | | | |

# **Table S7. Confidence intervals of Spearman's rank correlation coefficients at different time points**

| **Confidence Intervals of Spearman's Rho** | | | | | |
| --- | --- | --- | --- | --- | --- |
| Time | | **Spearman's rho** | **Significance**  **(2-tailed)** | **95% Confidence Intervals (2-tailed) ^a,b^** | |
|  |  |  |  | **Lower** | **Upper** |
| Before | BBS - TUG | -.820 | .000 | -.920 | -.621 |
|  | BBS - PGIC | .^c^ | . | . | . |
|  | BBS - MSWS-12 | -.440 | .022 | -.708 | -.060 |
|  | BBS - MSIS-29 | -.498 | .011 | -.752 | -.116 |
|  | TUG - PGIC | .^c^ | . | . | . |
|  | TUG - MSWS-12 | .642 | .000 | .328 | .828 |
|  | TUG - MSIS-29 | .732 | .000 | .456 | .879 |
|  | PGIC - MSWS-12 | .^c^ | . | . | . |
|  | PGIC - MSIS-29 | .^c^ | . | . | . |
|  | MSWS-12 - MSIS-29 | .822 | .000 | .630 | .919 |
| After 2 weeks | BBS - TUG | -.944 | .000 | -.981 | -.838 |
|  | BBS - PGIC | .219 | .368 | -.275 | .621 |
|  | BBS - MSWS-12 | -.748 | .000 | -.903 | -.419 |
|  | BBS - MSIS-29 | -.265 | .287 | -.660 | .244 |
|  | TUG - PGIC | -.360 | .155 | -.724 | .160 |
|  | TUG - MSWS-12 | .576 | .016 | .116 | .832 |
|  | TUG - MSIS-29 | .241 | .352 | -.286 | .656 |
|  | PGIC - MSWS-12 | -.353 | .151 | -.711 | .151 |
|  | PGIC - MSIS-29 | -.620 | .005 | -.843 | -.217 |
|  | MSWS-12 - MSIS-29 | .595 | .007 | .179 | .830 |
| After 4 weeks | BBS - TUG | -.860 | .000 | -.959 | -.574 |
|  | BBS - PGIC | .448 | .082 | -.077 | .779 |
|  | BBS - MSWS-12 | -.757 | .001 | -.913 | -.404 |
|  | BBS - MSIS-29 | -.551 | .022 | -.821 | -.080 |
|  | TUG - PGIC | -.210 | .452 | -.662 | .353 |
|  | TUG - MSWS-12 | .682 | .005 | .246 | .889 |
|  | TUG - MSIS-29 | .744 | .001 | .359 | .912 |
|  | PGIC - MSWS-12 | -.416 | .097 | -.754 | .097 |
|  | PGIC - MSIS-29 | -.181 | .473 | -.607 | .326 |
|  | MSWS-12 - MSIS-29 | .746 | .000 | .416 | .902 |
| After 8 weeks | BBS - TUG | -.757 | .049 | -.964 | .020 |
|  | BBS - PGIC | .372 | .324 | -.407 | .838 |
|  | BBS - MSWS-12 | -.826 | .002 | -.955 | -.433 |
|  | BBS - MSIS-29 | -.557 | .060 | -.862 | .044 |
|  | TUG - PGIC | .057 | .875 | -.608 | .675 |
|  | TUG - MSWS-12 | .778 | .014 | .214 | .953 |
|  | TUG - MSIS-29 | .633 | .067 | -.077 | .917 |
|  | PGIC - MSWS-12 | -.215 | .525 | -.732 | .458 |
|  | PGIC - MSIS-29 | -.005 | .989 | -.616 | .610 |
|  | MSWS-12 - MSIS-29 | .691 | .009 | .209 | .903 |
| After 12 weeks | BBS - TUG | -.690 | .040 | -.932 | -.023 |
|  | BBS - PGIC | -.099 | .800 | -.727 | .620 |
|  | BBS - MSWS-12 | -.478 | .162 | -.857 | .237 |
|  | BBS - MSIS-29 | -.185 | .610 | -.740 | .520 |
|  | TUG - PGIC | .017 | .965 | -.668 | .686 |
|  | TUG - MSWS-12 | .610 | .081 | -.114 | .911 |
|  | TUG - MSIS-29 | .600 | .088 | -.130 | .908 |
|  | PGIC - MSWS-12 | -.512 | .159 | -.883 | .253 |
|  | PGIC - MSIS-29 | -.641 | .063 | -.919 | .063 |
|  | MSWS-12 - MSIS-29 | .781 | .005 | .323 | .943 |
| a. Estimation is based on Fisher's r-to-z transformation. | | | | | |
| b. Estimation of standard error is based on the formula proposed by Fieller, Hartley, and Pearson. | | | | | |
| c. Cannot be computed because at least one of the variables is constant. | | | | | |
