# Supplementary material for: Potential efficacy of caffeine ingestion on balance and mobility in patients with multiple sclerosis: Preliminary evidence from a single-arm pilot clinical trial
Source: PLoS One. 2024 Feb 13;19(2):e0297235. doi: 10.1371/journal.pone.0297235 (PMC10863863; doi:10.1371/journal.pone.0297235)
Supplement: S2 File — (PDF) [file pone.0297235.s002.pdf]

وزارت بهداشت، درمان و آموزش پزشکی

دانشگاه علوم پزشکی زاهدان

معاونت تحقیقات و فناوری

طرح تحقیقاتی دانشجویی تا سقف

الف- اطلاعات کلی طرح

کد طرح	تاریخ تحویل به پژوهش دانشکده	تاریخ تصویب در شورای پژوهشی دانشکده/مرکز	تاریخ تصویب در شورای پژوهشی دانشگاه	تاریخ تصویب در کمیته اخلاق	تاریخ شروع	تاریخ خاتمه
۸۰۲۹	۱۳۹۵/۰۷/۰۸	۱۳۹۵/۰۸/۰۴	۱۳۹۶/۰۱/۱۸	۱۳۹۵/۰۹/۲۸	۱۳۹۶/۰۱/۱۸	۱۳۹۷/۰۱/۱۸

نام و نام خانوادگی مجری	زهرا رنجبر سیاهدشت.
عنوان طرح به فارسی	بررسی تاثیر مصرف کافئین بر راه رفتن و تعادل در بیماران مبتلا به مالتیپل اسکلروزیس در بیمارستان علی بن ابی طالب(ع) در سال ۱۳۹۵
عنوان طرح به انگلیسی	The effects of caffeine on walking and balance in patients with multiple sclerosis at Ali ibn Abi Talib (AS) in ۲۰۱۷
سه کلید واژه طرح/فارسی و انگلیسی(منطبق با MeSH)	تعادل- کافئین- مالتیپل اسکلروزیس
نوع طرح	کاربردی
محل اجرای طرح	بیمارستان علی ابن ابیطالب
بودجه کل(ریال)	۱۵,۰۰۰,۰۰۰
منابع تامین کننده بودجه طرح	بودجه پژوهشی
مدت زمان اجرا	۳۶۵

ب- اطلاعات مجری و همکاران طرح

ردیف	نام و نام خانوادگی	سمت در طرح	نوع همکاری	مقطع تحصیلی	رشته تحصیلی	توضیحات
۱	زهرا رنجبر سیاهدشت	مجری	تدوین طرح	پزشکی عمومی	پـزشکـــــــــی	
۲	فرزانه فرجیان مشهدی	مشاور	مشاور تخصصی	دکترای تخصصی	فارماکولوژی	
۳	حسین انصاری	مشاور	مشاور آمار	دکترای تخصصی	اپیدمیولوژی	

۴	افسون دادور	همکار	انجام مراحل بالینی	پزشکی عمومی	پـزشکـــــــــــی	
۵	علیرضا خسروی	استاد راهنما	تدوین طرح	دکترای تخصصی	نورولوژی	

پ- اطلاعات مربوط به طرح پژوهشی:

۱	**خلاصه طرح به فارسی :**<br><br>بررسی تاثیر مصرف کافئین بر راه رفتن و تعادل در بیماران مبتلا به مالتیپل اسکلروزیس در بیمارستان علی بن ابی طالب(ع) در سال ۱۳۹۵
۲	**خلاصه طرح به لاتین :**<br><br>The effects of caffeine on walking and balance in patients with multiple sclerosis at Ali ibn Abi Talib (AS) in ۲۰۱۷
۳	**بیان مسئله تحقیق (مشتمل بر مرور تحقیقات گذشته داخلی و خارجی مرتبط با موضوع با ذکر منابع و توجیه ضرورت انجام طرح) :**<br><br>مالتیپل اسکلروزیس شایعترین علت ناتوانی عصبی در جوانان است.وسعت این بیماری در جهان امروز بسیار بیشتر شده به گونه ای که هم اکنون حدود ۲.۵ میلیون نفر در جهان به این بیماری مبتلا میباشند (۱)از جمله علائم این بیماری میتوان به خستگی زودرس،اختلالات حسی،درد،نقص در توان حرکتی و تعادل،اختلال شناختی و علائم بینایی اشاره کرد (۱)مطالعات نشان داده است ژنتیک و هم چنین عوامل محیطی از جمله عوامل تاثیر گذار برروی ابتلا به بیماری MSمی باشند (۲)این بیماران به دلیل اختلالات شناختی دچار مشکلاتی در سرعت پردازش،توجه،عملکرد اجرایی،یادگرفتن و حافظه میشوند(۳).ازجمله ریسک فاکتور های این بیماری میتوان کنترل وضعی نامناسب و ضعیف،ناتوانی،ترس از افتادن،مشکلات راه رفتن،مشکلات حسی و استفاده از وسایل کمکی را نام برد(۳).که نیروی عضلانی و عملکرد حرکتی را جزء ریسک فاکتور های قدیمی و عملکرد شناختی را جزء ریسک فاکتورهای جدید شناخته شده دسته بندی کرده اند(۳).<br><br>اختلال حرکتی یکی از نتایج شایع این بیماری ست (۴, ۵) وشاید گاهی اولین علامت آن باشد(۶) فامپریدین دارویی ست که برای پیشرفت راه رفتن و افزایش سرعت آن به کار میرود(۷, ۸). این دارو پیشرفت دائمی را در سرعت راه رفتن بیماران موجب میگردد(۹).<br><br>راه رفتن فرآیند پیچیده ایست که عوامل بسیاری برروی آن تاثیر میگذارند که یکی از آن ها اختلال در تعادل میباشد (۱۰, ۱۱) که در بین بیماران مالتیپل اسکلروزیس به فراوانی دیده شده است (۱۲)و دریافته اند که آن موجب کاهش سرعت راه رفتن بیماران ام اس میگردد (۱۰)و در نتیجه باعث بسیاری از مشکلات برای این بیماران میشود.<br><br>در نتیجه برای آنکه بتوانیم توانایی راه رفتن را بهبود ببخشیم ابتدا باید به صورت ریشه ای به بهبود تعادل این بیماران بپردازیم.<br><br>کافئین یک آلکالوئید تلخ و بدون بو و بلورین و سفیدرنگ با فرمول $C_8H_{10}N_4O_2$ میباشد که در نوشیدنی هایی هم چون چای و قهوه و کولا و نوشابه و های گازدار و نوشابه های انرژی زا وجود دارد (۱۳-۱۵). کافئین در انسان موجب تحریک سیستم عصبی

مرکزی میشود (۱۴, ۱۶)هم چنین از دیگر اثرات آن میتوان به۱)سهولت انتقال پیام های عصبی دستگاه عصبی سمپاتیک(۱۷)،۲)افزایش انرژی مصرفی استراحت(متابولیسم پایه) (۱۸)،۳)افزایش استقامت و بهبود عملکرد(۱۹)،۴)تقویت عملکرد شناختی (۲۰)،۵)افزایش انرژی ذهنی (۱۸, ۲۱)۶)افزایش هماهنگی عصبی-عضلانی (۲۲, ۲۳)، اشاره کرد. این ماده علاوه بر تاثیری که در تولید انرژی دارد موجب افزایش غلظت سروتونین در ساقه ی مغز می شود (۱۹)که این محرک نورون های حرکتی نخاع،موجب تحریک واحد های حرکتی عضلات اسکلتی میگردد (۱۹)علاوه بر آن مصرف یک دوز کوچک حدود ۳۲-۵۰ میلی گرم کافئین یک محرک قوی برای تقویت هوشیاری و توانایی تمرکز در کمتر از ۲۰ دقیقه می باشد (۲۴)حتی پس از ۵۰ دقیقه از مصرف ۱۴۰ میلی گرم آن میتوان افزایش قابل توجهی در هوشیاری ذهنی افراد مشاهده نمود (۲۵).

میزان کافئین لازم برای بروز اثرات تحریکی آن به اندازه ی جثه ی بدن و میزان تحمل افراد بستگی دارد و در افراد مختلف میزان مصرف آن متفاوت است. مصرف معمول کافئین تا ۹۰۰میلی گرم در روز برای سلامتی خطر ندارد اما نباید به صورت یکباره مصرف شود و باید به سه دوز ۳۰۰ میلی گرم در روز تقسیم گردد.(۲۶)معمولا ظرف مدت ۱۵-۴۵ دقیقه مقادیر این ماده در خون بالا رفته و طی یک ساعت به حداکثر غلظت خود در خون میرسد کمتر از یک ساعت اثرات این ماده ظاهر و پس از ۵ ساعت اثر آن از بین میرود(۱۷, ۲۷).مطالعات گذشته نگر ارتباطی را بین مصرف ۳ فنجان یا بیشترقهوه را در روز با افزایش بیماری قلبی عروقی مطرح میسازند اما مطالعات آینده نگر هیچ گونه ارتباطی بین هر مقدار مصرف این ماده و بیمایر قلبی را تایید نمیکند(۲۸, ۲۹). نکاتی که باید در رابطه با مصرف کافئین مد نظر قرار گیرد عبارتند از:۱)گروهی از افراد به دلیل مصرف کافئین در شب دچار بی خوابی میشوند.۲)مصرف این ماده در افرادی که عادت به مصرف ندارند می تواند مدر باشد.۳)این ماده ممکن است موجب تشدید علائم میگرن یا کهیر و یا اختلالات گوارشی گردد.(۳۰)در رابطه با ایجاد اعتیاد ناشی از مصرف کافئین هم مطالعات قبلی نشان داده است که تنها در صورت کاهش ناگهانی بیشتر از ۱۰۰ میلی گرم در روز امکان بروز علائم ترک کافئین در افراد وجود دارد(۳۱) در مطالعه ای دیگر تیجه گیری شده است که استفاده از نوشیدنی های کافئین دار هیچ ارتباطی با سندروم restless leg ندارد(این سندروم موسوم به RLS نوعی اختلال حرکتی عصبی در بیماران مبتلا به MS می باشد) (۳۲)و نکته ی دیگر اینکه مصرف کافئین هیچ تاثیری برروی ریسک بیماری MS ندارد(۳۳)حتی مطالعات قبلی نشان داده اند که ریسک ابتلا به بیماری مالتیپل اسکلروزیس در بین افرادی که روزانه مقدار زیادی قهوه (۹۰۰ میلی لیتر در روز)مصرف میکنند کاهش می یابد(۳۴). همچنین مطالعات قبلی برروی موش ها نشان داده است که با توجه به این که کافئین آنتاگونیست گیرنده آدنوزین است در نتیجه میتواند از انسفالو میلیت ناشی از بیماری خودایمن مالتیپل اسکلروزیس جلوگیری کرده وهمچنین نتیجه ی این مقاله بدین گونه است که مصرف کافئین به مدت طولانی ممکن است آسیب های مغزی در MS را کاهش دهد.(۳۵))

از آنجایی که دارویی که برای بهبود راه رفتن در بیماران مبتلا به ام اس مورد استفاده قرار میگیرد نیازمند هزینه ی بسیار بالاست و ماده ی کافئین موجب بهبود عملکرد سیستم عصبی مرکزی و در نتیجه افزایش هماهنگی عصب عضله میگردد و از آنجا که هیچگونه مطالعه ای در این رابطه تا به کنون انجام نگرفته است، برآن شدیم که تاثیر کافئین را برروی عملکرد حرکتی بیماران مبتلا به مالتیپل اسکلروزیس بسنجیم.

۴	**سوالات و/یا فرضیات پژوهش :** <br>**آیا میانگین میزان توانایی راه رفتن و تعادل در بیماران مبتلا به مالتیپل اسکلروزیس در زمان دریافت پلاسبو و کافیین متفاوت است؟**

میانگین امتیاز بدست آمده از تست EDSSدر افراد مبتلا به مالتیپل اسکلروزیس با دریافت پلاسبو چقدر است؟

میانگین امتیاز بدست آمده از تستEDSS در افراد مبتلا به مالتیپل اسکلروزیس با دریافت کافیین چقدر است؟

میانگین امتیاز بدست آمده از تستBBS در افراد مبتلا به مالتیپل اسکلروزیس با دریافت پلاسبو چقدر است؟

میانگین امتیاز بدست آمده از تستBBS در افراد مبتلا به مالتیپل اسکلروزیس با دریافت کافیین چقدر است؟

میانگین امتیاز بدست آمده از تست۱۲-MSWS در افراد مبتلا به مالتیپل اسکلروزیس با دریافت پلاسبو چقدر است؟

میانگین امتیاز بدست آمده از تست۱۲-MSWS در افراد مبتلا به مالتیپل اسکلروزیس با دریافت کافیین چقدر است؟

میانگین امتیاز بدست آمده از تست۲۹-MSIS در افراد مبتلا به مالتیپل اسکلروزیس با دریافت پلاسبو چقدر است؟

میانگین امتیاز بدست آمده از تست۲۹-MSIS در افراد مبتلا به مالتیپل اسکلروزیس با دریافت کافیین چقدر است؟

میانگین میزان زمان لازم برای انجام تست TUG در افراد مبتلا به مالتیپل اسکلروزیس با دریافت پلاسسبو چقدر است؟

میانگین میزان زمان لازم برای انجام تست TUG در افراد مبتلا به مالتیپل اسکلروزیس با دریافت کافیین چقدر است؟

میانگین امتیاز بدست آمده از تستPGICدر افراد مبتلا به مالتیپل اسکلروزیس با دریافت پلاسبو چقدر است؟

میانگین امتیاز بدست آمده از تست**PGIC**در افراد مبتلا به مالتیپل اسکلروزیس با دریافت کافئین چقدر است؟<br><br>میانگین امتیاز بدست آمده از تست**EDSS** در افراد مبتلا به مالتیپل اسکلروزیس در زمان استفاده از پلاسبو با زمان دریافت کافئین تفاوت معنادار دارد.<br><br>میانگین امتیاز بدست آمده از تست**PGIC** در افراد مبتلا به مالتیپل اسکلروزیس در زمان استفاده از پلاسبو با زمان دریافت کافئین تفاوت معنادار دارد.<br><br>میانگین امتیاز بدست آمده از تست**BBS** در افراد مبتلا به مالتیپل اسکلروزیس در زمان استفاده از پلاسبو با زمان دریافت کافئین تفاوت معنادار دارد.<br><br>میانگین امتیاز بدست آمده از تست**MSWS**-۱۲ در افراد مبتلا به مالتیپل اسکلروزیس در زمان استفاده از پلاسبو با زمان دریافت کافئین تفاوت معنادار دارد.<br><br>میانگین امتیاز بدست آمده از تست**MSIS**-۲۹ در افراد مبتلا به مالتیپل اسکلروزیس در زمان استفاده از پلاسبو با زمان دریافت کافئین تفاوت معنادار دارد.<br><br>میانگین زمان لازم برای انجام تست **TUG** در افراد مبتلا به مالتیپل اسکلروزیس در زمان استفاده از پلاسبو با زمان دریافت کافئین تفاوت معنادار دارد.<br><br>مقایسه میزان توانایی راه رفتن و تعادل در بیماران مبتلا به مالتیپل اسکلروزیس در زمان دریافت پلاسبو و کافیین		
هدف کلی طرح :<br><br>تعیین **تاثیر مصرف کافئین بر توانایی راه رفتن و تعادل در بیماران مبتلا به مالتیپل اسکلروزیس مراجعه کننده به کلینیک نورولوژی بیمارستان امام علی در سال ۹۵-۹۶**	۵
اهداف اختصاصی :	۶

الف)تعیین میانگین امتیاز آزمون EDSSدر افراد مبتلا به مالتیپل اسکلروزیس در گروه دریافت پلاسبو.

ب)تعیین میانگین امتیاز آزمون EDSS در افراد مبتلا به مالتیپل اسکلروزیس در گروه دریافت کافئین

ج) تعیین میانگین امتیاز آزمون ۱۲-MSWSدر افراد مبتلا به مالتیپل اسکلروزیس در گروه دریافت پلاسبو.

د) تعیین میانگین امتیاز آزمون ۱۲-MSWSدر افراد مبتلا به مالتیپل اسکلروزیس در گروه دریافت کافئین

ح)تعیین میانگین امتیاز آزمون BBS در افراد مبتلا به مالتیپل اسکلروزیس در گروه دریافت پلاسبو

ه) تعیین میانگین امتیاز آزمون BBS در افراد مبتلا به مالتیپل اسکلروزیس در گروه دریافت کافئین

ی)تعیین میانگین امتیاز آزمون ۲۹-MSIS در افراد مبتلا به مالتیپل اسکلروزیس در گروه دریافت پلاسبو

پ) تعیین میانگین امتیاز آزمون ۲۹-MSIS در افراد مبتلا به مالتیپل اسکلروزیس در گروه دریافت کافئین

خ)تعیین میانگین زمان لازم برای انجام تست TUG در افراد مبتلا به مالتیپل اسکلروزیس در گروه دریافت پلاسبو

ث) تعیین میانگین زمان لازم برای انجام تست TUG در افراد مبتلا به مالتیپل اسکلروزیس در گروه دریافت کافئین

ع) تعیین میانگین امتیاز آزمون PGICدر افراد مبتلا به مالتیپل اسکلروزیس در گروه دریافت پلاسبو

غ) تعیین میانگین امتیاز آزمون PGIC در افراد مبتلا به مالتیپل اسکلروزیس در گروه دریافت کافئین

۸	**سازمان ها، مراکز و موسسات سایر بهره برداران بالقوه دستاوردهای طرح :**
۹	**تعریف واژه های تخصصی :**<br><br>مالتیپل اسکلروزیس شایعترین علت ناتوانی عصبی در جوانان است وسعت این بیماری در جهان امروز بسیار بیشتر شده به گونه ای که هم اکنون حدود ۲.۵ میلیون نفر در جهان به این بیماری مبتلا میباشند از جمله علائم این بیماری میتوان به خستگی زودرس،اختلالات حسی،درد،نقص در توان حرکتی و تعادل،اختلال شناختی و علائم بینایی اشاره کرد

نکته:درصورتی که رسته مطالعاتی شما کیفی می باشد نیاز به تکمیل جدول متغیرها نیست.

نام متغیر	نقش متغیر	نوع متغیر	تعریف کاربردی	مقیاس متغیر
زمان	مستقل	کیفی –اسمی	ابتدا در طی یک ماه داروهای ملازم و پلاسبو را دریافت میکنند سپس به مدت ۳ ماه داروهای ملازم و کافئین را دریافت مینمایند.	قبل و بعد از دریافت کافئین
آزمون EDSS	وابسته	کمی –پیوسته	براساس نتایج آزمون Kurtzke Expanded Disability Status Scale ضمیمه شده حداقل=۰ حداکثر=۱۰	امتیاز
آزمون ۱۲–MSWS	وابسته	کمی– گسسته	براساس نتایج آزمون Twelve Item MS Walking Scale ضمیمه شده حداقل=۱ حداکثر=۶۰	امتیاز
آزمون PGIC	وابسته	کمی– گسسته	براساس نتایج آزمون Patients' Global Impression of Change ضمیمه شده حداقل=۱ حداکثر=۷	امتیاز
آزمون TUG	وابسته	کمی– گسسته	طبق دستور آزمون تحت نظر کارشناس انجام میگردد و زمان به وسیله ی کورنومتر اندازه گیری میگردد. براساس نتایج آزمون The Timed Up and Go (TUG) Testضمیمه شده	ثانیه
آزمون BBS	وابسته	کمی– گسسته	براساس نتایج آزمون Berg Balance Scaleضمیمه شده حداقل=۰ حداکثر=۵۶	امتیاز
آزمون ۲۹–MSIS	وابسته	کمی– گسسته	براساس نتایج آزمون Multiple Sclerosis Impact Scale ضمیمه شده حداقل=۰ حداکثر=۱۰۰	امتیاز

ت– اطلاعات مربوط به روش اجرای طرح

۱	**نوع مطالعه:**<br><br>کارآزمایی بالینی

۲ جامعه مورد مطالعه (معیارهای ورود و خروج ذکر شوند):

بیماران مبتلا به مالتیپل اسکلروزیس مراجعه کننده به بیمارستان علی بن ابیطالب زاهدان

۳ در صورت نمونه گیری حجم نمونه و روش محاسبه آن:

حجم نمونه ۳۰ نفر از افراد دارای MSحاضر در بیمارستان علی بن ابیطالب زاهدان

حجم نمونه بر اساس رفرنس(۳۶, ۳۷) و حجم در دسترس در بیمارستان علی بن ابیطالب وارد مطالعه خواهند شد.

۴ روش نمونه گیری (نحوه تخصیص تصادفی و همسان سازی در صورت لزوم ذکر شود):

نمونه گیری به روش غیراحتمالی در دسترس مورد بررسی قرار خواهند گرفت و با استفاده از بلوک های تصادفی تصادفی سازی صورت خواهد گرفت و همچنین همسان سازی از لحاظ سن ، وزن بالای ۴۰ کیلوگرم ،عدم وجود آلرژی یا ابتلا به بیماری خاص و شروع تشدید علائم بیماری ام اس از ۶۰ روز قبل از غربالگری نیز به صورت گروهی صورت خواهد پذیرفت.

۵ روش و ابزار جمع آوری داده ها و اطلاعات (پرسشنامه یا فرم اطلاعاتی ضمیمه شود):

براساس آزمون **TUG** و **BBS** و **PGIC** و۱۲-**MSWS** و **EDSS** اطلاعات بیماران یکبار پس از دادن پلاسبو و بار دیگر پس از دادن کافئین در هفته های ۲-۴-۸و۱۲اندازه گیری و ثبت میگردد.

۶ روش اجرای طرح (با جزئیات بطور کامل شرح داده شود):

افراد با تشخیص قطعی مالتیپل اسکلروزیس وارد مطالعه می شوند .

از افراد مورد مطالعه پس از آموزش در مورد نحوه انجام کار رضایتنامه ی کتبی گرفته میشود سپس بیماران براساس شایستگی شرکت در آزمایش غربال می گردند. در روز اول تصادفی سازی و همسان سازی و همچنین تهیه اسناد صورت می پذیرد همچنین اطلاعات دموگرافیک مانند ، سن و جنس و میزان ناتوانی بر اساس آزمون (**EDSS(expanded disability status scale score** که براساس امتیازهای ۰-۱۰ که میزان پیشرفتگی بیماری را نیز تشخیص می دهد،ثبت میشوند.در ماه اول به بیماران طبق معمول پلاسبو داده میشود و در پایان تمامی تست ها انجام شده و نتایج ثبت و بایگانی میگردد سپس به همان بیماران در دوره ی سه ماهه ۲.۵ میلی گرم به ازای هر کیلوگرم وزن بیمار(کمترین دوز ممکن)به صورت روزانه داده میشود و در روز اول و هفته های ۲،۴،۸،۱۲،۱۶،۲۰،۲۴ طبق برنامه ویزیت میگردد.لازم به ذکر است که در طی این مدت مصرف مواد خوراکی کافئین دار توسط بیمار محدود میگردد.

توان راه رفتن بیماران براساس آزمون (**item ms walking scale**(۱۲-۱۲**MSWS** (پرسشنامه ایست که از ۱۲ سوال تشکیل شده است که میزان محدودیت حرکت در این بیماران را در طی دو هفته پاسخ گویی آن ها نشان میدهد.هر یک از سوال های این آزمون بر اساس ۱-۵ امتیاز بندی شده اند و جمع کل امتیازهای آن ۱-۶۰ میباشد که کاهش امتیاز در این آزمون نشانگر پیشرفت در راه رفتن بیماراست.) آزمون **PGIC** (ارزیابی خود بیمار از تاثیر داروی مصرف شده در طی هفت روز گذشته میباشد و براساس امتیازهای ۱-۷ برنامه ریزی شده که هر چه امتیاز بیشتر باشد میزان تاثیر دارو بیشتر بوده است.)نیز در هفته های ۲،۴،۸،۱۲،۱۶،۲۰،۲۴توسط خود بیمار ارزیابی میگردد.

همچنین تحرک و تعادل حرکتی بیماران براساس تست **TUG**(**TIME UP AND GO**((به معنی اندازه گیری سرعت-زمان میباشد بدین صورت که بیمار از صندلی بلند می شود و می ایستد ، به اندازه سه متر راه میرود و سپس میچرخد و به سمت مکان اولیه اش بازمیگردد و برروی صندلی اش مینشیند که افزایش سرعت در این آزمون نشانگر پیشرفت تحرک و تعادل حرکتی بیمار میباشد.) و تعادل ایستا و

[illegible]

حرکتی براساس تست BBS(berg balance scale) (که از ۱۴ حرکت تعادلی تشکیل شده است و هریک براساس ۰-۴ امتیازدهی میگردند و در مجموع امتیاز های ۰-۵۶ حاصل میگردند که تغییرات مثبت در این آزمون نشان دهنده پیشرفت تعادل بیمار میباشد.)

میزان پیشرفتگی بیماری و کیفیت زندگی نیز براساس MSIS-۲۹ (از ۲۰ آیتم مربوط به مقیاس فیزیکی و ۹ آیتم مربوط به مقیاس های روحی روانی تشکیل شده است و از ۰-۱۰۰ امتیاز بندی میگردند در صورت کاهش امتیاز در مقیاس فیزیکی پیشرفت سلامت فیزیکی نتیجه میگردد.)به صورت مکرر و همراه با دیگر آزمون ها انجام خواهد گرفت.

بی خطری و تحمل بیماران را براساس AEs (ملاحظه عوارض جانبی)و SAEs(ملاحظه عوارض جانبی خطرناک) و داروهای ملازم و آزمون های فیزیکی و بررسی علائم حیاتی و تهیه الکتروکاردیو گرام سنجیده میشود.

در نهایت براساس نتایج حاصل از آزمون های فوق نتیجه نهایی گزارش میگردد ضمنا جهت کاهش علائم ناشی از ترک کافئین در بیماران در انتها به آن ها آموزش داده خواهد شد که میزان مصرف مواد خوراکی حاوی کافئین را تا مدتی ادامه دهند.

۷ **روش تجزیه تحلیل و توصیف داده ها (نمونه ای از جدول توخالی ضمیمه شود و روش های آماری مورد استفاده به طور کامل توضیح داده شود):**

با استفاده از روش های آماری توصیفی(تنظیم جداول توزیع فراوانی، تعیین شاخص های مرکزی و پراکندگی)و آمار تحلیلی(آزمون T زوجی و در صورت نرمال نبودن آن ویلکاکسون)داده ها در سطح اطمینان ۹۵ درصد و بااستفاده از نرم افزار SPSS تجزیه و تحلیل خواهند شد.

۸ **ملاحظات اخلاقی (در صورت لزوم فرم رضایت آگاهانه ضمیمه گردد.درج کدهای اخلاقی منطبق با کدهای مصوب حفاظت از آزمودنی در پژوهش های علوم پزشکی الزامی است):**

کارآزمایی‌بالینی معتبرترین روش تهیه‌ی مستندات برای استفاده‌ی بالینی است. انجام کارآزمایی بالینی برای پیشرفت علوم پزشکی از ضرورت و اهمیت ویژه‌ای برخوردار است. اما در عین حال، نظر به این‌که در کارآزمایی‌های بالینی، بر خلاف سایر انواع مطالعات، مداخله‌ای به‌صورت عمدی بر روی آزمودنی‌ها به انجام می‌رسد، این شیوه‌ی پژوهش، بیش از سایر انواع و شیوه‌ها، دربردارنده‌ی ملاحظات و دغدغه‌های اخلاقی است.

این راهنما دربردارنده‌ی مهم‌ترین ملاحظات اخلاقی است که در این زمینه باید رعایت شوند. هر پژوهشگر یا بالینگری که در کارآزمایی بالینی مشارکت می‌کند، باید علاوه بر مفاد این راهنما، از راهنمای عمومی اخلاق در پژوهش‌های علوم پزشکی، راهنماهای اختصاصی اخلاق در پژوهش کشور به فراخور موضوع، و نیز از سایر قوانین و مقررات مرتبط با کار خود آگاه باشد و آن‌ها را رعایت کند. این موضوع به‌ویژه در رابطه با رعایت مفاد راهنمای پژوهش بر گروه‌های آسیب‌پذیر مورد تأکید است.

فصل اول: ارزیابی سود و زیان

۱. کارآزمایی‌های بالینی باید کاملاً درچارچوب یک طرح‌نامه و دستورالعمل مکتوب طراحی و اجرا شوند. طرحنامه و دستورالعمل کارآزمایی بالینی باید دربردارنده‌ی بخشملاحظات اخلاقی، هم‌چنین اطلاعات مربوط به بودجه‌ی پژوهش، حامیان پژوهش، وابستگیحرفه‌ای، بیان هرگونه تعارض منافع احتمالی وتمهیدات مورد نظر برای ترغیب مشارکت افراددر مطالعهباشد.
۲. شروع اجرای کارآزمایی منوط به بررسی و تأیید طرح‌نامه و دستورالعمل آن توسط کمیته‌ی اخلاق در پژوهشاست. کمیته‌ی اخلاق درپژوهش حق پایش کارآزمایی‌های در حال اجرا را دارد. پژوهشگر موظف است که اطلاعاتمورد نیاز برای پایش را در اختیار کمیته قراردهد.
۳. پژوهشگر موظف است که در طول اجرای پژوهش، هرگونه حادثه یا عارضه‌ی نامطلوب جدی قابل انتساب به پژوهش را در اولین زمان ممکن، به کمیته‌ی اخلاق در پژوهش و سایر مراجع قانونی ذی‌ربط گزارش دهد.
۴. کمیته‌ی اخلاقمسؤولیت دایمی نظارت بر اجرای اخلاقی پژوهش را بر عهده دارد، لذا پژوهشگر ارشد باید این کمیته را در موردتمامی تغییرات دستورالعمل مطالعه و هر حادثه‌ی نامناسب جدی در طول مطالعه آگاهسازد. هم‌چنین، هر اطلاعات جدیدی که ممکن است امنیت آزمودنی یا اجرایمطالعه را تحت تأثیر قرار دهد را باید به اطلاع این کمیته برساند.
۵. کارآزمایی بالینی بایدتنها توسط افراد دارای مجوز حرفه‌ای مرتبط و ذی‌صلاح از نظر علمي انجام گیرد.
۶. انجام کارآزمایی بالینی تنهازمانی قابل توجیه است که جامعه‌ای که افراد تحت مطالعه به آن تعلق دارند بتوانند ازنتایج آن پژوهش سود ببرند.

۷. تمامی اقدامات احتیاطی لازم جهت حفظ حریم خصوصیآزمودنی‌ها، محرمانه ماندن اطلاعات مربوط به ایشان و هم‌چنین کاهش تأثیر نامطلوبمطالعه بر سلامت جسمی و روانی آزمودنی‌ها باید به‌عمل آید.
۸. در مرحله‌ی طراحی مطالعه، باید نحوه‌ی پی‌گیریآزمودنی‌ها پس از اتمام مطالعه تعیین شود و در صورت لزوم، برای دسترسی آن‌ها به بهترین روش پیش‌گیری، تشخیص، درمان یا سایر مراقبت‌های مناسب، تمهیدات لازم درنظر گرفته شود. دسترسی لزوماً به معنی فراهم آوردن خدمات رایگان نیست.
۹. در صورت وقوع عوارض یاحوادث نامطلوب قابل انتساب به پژوهش، درحین و پس از مطالعه، پژوهشگر باید اقدامات درمانی و مراقبتی مناسب را برایآزمودنی، بدون تحمیل هزینه به وی، فراهم آورد. تمهیدات مالی برای انجام این تعهد، نظیر بیمه کردن پژوهش، باید در هنگام طراحی مطالعه در نظر گرفته شده باشد.
۱۰. اگر در حین یا بعد از انجام پژوهش، وجود بیماری یا وضعیت مرتبط با سلامت خاصی در آزمودنی تشخیص داده شد، پژوهشگر یا مؤسسه‌ی حامی باید در صورت آزمودنی، وی را از این موضوع آگاه کند.
۱۱. پژوهشگرموظف است که در صورت رضایت آزمودنی، شرکت او در کارآزمایی را به اطلاع پزشک خانواده‌ی وی برساند.
۱۲. کلیه‌ی اطلاعات کارآزمایی بالینی باید به‌گونه‌ای ثبت، به‌کارگیری و ذخیره شود که امکان شناسایی، گزارش و تفسیر دقیق آن‌ها فراهم باشد.
۱۳. هرگونه پرداخت مالی به آزمودنی باید تنها در محدوده‌ی بازپرداخت هزینه‌های تحمیل‌شده به وی در اثر شرکت در پژوهش و قدردانی از او باشد. باید از هرگونه پرداخت غیرمتعارف – که احتمال داشته باشد که آزادی فرد برای قبول یا تداوم مشارکت در پژوهش را خدشه‌دار کند – خودداری شود.
۱۴. مطالعات دوسوکور باید به‌گونه‌ای طراحی شوند که در صورت وقوع عارضه‌ای برای هرکدام از آزمودنی‌ها که شکستن کد را ایجاب کند، فردی که امکان شکستن کد را برای آن آزمودنی دارد و نحوه‌ی انجام این کار مشخص باشد. جزئیات این موضوع باید در دستورالعمل کارآزمایی آورده شود.
۱۵. هیچ مداخله‌ای که هنوز بر اساس پزشکی مبتنی بر شواهد تأیید نشده است، نباید به دلایلی نظیر گیاهی یا سنتی بودن از طی تمامی مراحل استاندارد آزمون و کارآزمایی مستثنی شمرده شود.
۱۶. چنان‌چه برای یک کارآزمایی بالینی فاز یک، آزمودنیزن مورد نیاز باشد، باید اینافراد در سن باروری نباشند یا از روش‌های قطعی پیشگیری از بارداری استفادهکنند.
۱۷. در کارآزمایی‌های با پرتوتابی، نوع و دوز مداخله بایدبه تأیید کمیته‌ی اخلاق رسیده باشد. این تأییدیه نیز باید بر اساس نظر مشورتی تخصصی باشد.
۱۸. داوطلبان سالم در کارآزمایی‌های با پرتوتابی باید بیشاز ۵۰ سال سن داشته باشند. در صورتی می‌توان از افراد با سن کم‌تر استفاده کرد کهمطالعه مربوط به گروه سنی ایشان باشد. تعداد شرکت‌کنندگان باید در حداقل تعداد ممکنبا توجه به هدف مطالعه و دقت مورد نیاز انتخاب شود.
۱۹. در صورتی‌که مداخله‌ی دارویی در مطالعه مد نظر باشد و داروی مورد نظر در فهرست دارویی کشور وجود نداشته یا به ثبت نرسیده باشد، فرایند صدور مجوز انجام مطالعه‌ی بالینی و هم‌چنین واردات و ترخیص داروی تحقیقاتی مورد استفاده در کارآزمایی بالینی تابع مقررات و ضوابط مربوطه و به‌عهده‌ی سازمان غذا و دارو است.

فصل دوم: رضایت آگاهانه

۱. اخذ رضایت آگاهانه برای کارآزمایی بالینی باید همواره به‌صورت کتبی باشد. فرم رضایت‌نامه باید دربردارنده‌ی تمامی اطلاعات لازم برای تصمیم‌گیری فرد جهت شرکت یا عدم شرکت در پژوهش – شامل اطلاعاتی که در بندهای بعدی این راهنما ذکر شده اند – باشد.
۲. فرم رضایت آگاهانه باید توسط پژوهشگر ارشد – یا عضو دیگری از تیم پژوهشی که آگاهی و توانایی لازم را دارد، به‌عنوان نماینده‌ی پژوهشگر ارشد – و آزمودنی یا نماینده‌ی قانونی او امضا شود. این فرم باید حداقل در دو نسخه تهیه شود که یک نسخه‌ی آن به آزمودنی تحویل داده می‌شود و نسخه‌ی دیگر باید توسط پژوهشگر نگه‌داری شود.
۳. در مواردی که فرد به هر دلیلی قادر به خواندن فرم رضایت‌نامه‌ی مکتوب نباشد، باید فرد ثالثی که دارای تعارض منافع نباشد، مندرجات فرم را به زبان قابل فهم برای آزمودنی توضیح داده، به پرسش‌های او پاسخ دهد. در این حالت، فرم باید علاوه بر امضای پژوهشگر و امضا یا اثر انگشت آزمودنی، واجد امضای فرد ثالث پیش‌گفته نیز باشد
۴. هنگام ارسال طرح‌نامه برای بررسی توسط کمیته‌ی اخلاق، فرم رضایت‌نامه‌ای که قرار است به آزمودنی‌ها ارائه شود، باید به طرح‌نامه پیوست باشد. بررسی اخلاقی طرح‌نامه‌ی کارآزمایی بالینی بدون بررسی و ارزیابی فرم رضایت آگاهانه‌ی آن معتبر نخواهد بود.
۵. برای اخذ رضایت، اطلاعات باید به زبانی ارائه شود کهبرای آزمودنی قابل فهم باشد. آزمودنی یا نماینده‌ی قانونی او باید فرصتکافی برای پرس‌وجو در مورد جزییات کارآزمایی را داشته باشند. باید به‌طور مشخصاعلام شود که کارآزمایی یک فرایند پژوهشی است که مشارکت در آن داوطلبانه است و عدمقبول شرکت یا خارج شدن از کارآزمایی در هر زمانی، مراقبت از نمونه، حقوق و سلامت ویرا تحت تأثیر قرار نخواهد داد.
۶. آزمودنی باید به اطلاعات در موردبیمه و سایر تمهیدات برای جبران صدمات ناشی از مشارکت در کارآزمایی دسترسی داشتهباشد. هم‌چنین، وی باید در مورد درمان‌هایی که در صورت بروز صدمه یا ناتوانی بهدنبال شرکت در کارآزمایی، در اختیار وی قرار خواهد گرفت آگاه شود
۷. اخذ رضایت آگاهانه فرایندی است که از آغاز تا پایان ارتباط پژوهشگر–آزمودنی تداوم دارد. هر زمان‌که اطلاعات جدیدی به‌دست آید که امکان داشته باشد که در تصمیم‌گیری آزمودنی‌ها جهت قبول یا تداوم شرکت در پژ. وهش تأثیرگذار باشد، این اطلاعات باید

به‌صورت مکتوب در اختیار آزمودنی‌ها قرار گیرد.

۸. در زمان اخذ رضایت، باید احتیاط شود که آزمودنی‌ها رضایت خود راتحت محذوریت و به‌علت وابستگی درمانی، اداری و. . . نداده‌باشند. در مواردی که این احتمال وجود دارد، رضایت‌آگاهانه باید توسط فرد دیگری که اطلاع کافیاز مطالعه دارد و در عین حال چنین رابطه‌ای با آزمودنی ندارد، کسب شود.
۹. شروع و تداوم شرکت آزمودنی در پژوهش باید آزادانه باشد. از همین رو، هیچ‌یک از اعضای تیم پژوهش نباید آزمودنی‌ها را برای ادامه‌ی مشارکت در مطالعه مورد اجبار، تطمیع، اغوا، تهدید و/ یاتحت محذوریت قرار دهند.
۱۰. از آن‌جا که تمامی عوارض و خسارات قابل انتساب به پژوهش برای آزمودنی‌ها باید جبران شود، اخذ برائت ذمه هیچ جایگاهی در کارآزمایی بالینی ندارد و نباید در فرم رضایت آگاهانه گنجانده شود. این امر رضایت آگاهانه‌ی پژوهشی را از رضایت‌نامه‌ی درمانی متمایز می‌کند.
۱۱. فرم رضایت آگاهانه که برای اخذ رضایت به آزمودنی داده می‌شود، باید دربردارنده‌ی اطلاعات ذیل باشد:

۱. عنوان کارآزمایی
۲. ماهیت پژوهشی کارآزمایی
۳. هدف کارآزمایی
۴. درمان (یامداخله) در کارآزمایی و احتمال تخصیص تصادفی به هر درمان یا مداخله
۵. روش‌های پی‌گیری شامل روش‌های تهاجمی و غیرتهاجمی
۶. مسؤولیتآزمودنی‌ها
۷. جنبه‌هایی از کارآزمایی که ماهیت پژوهشی دارد.
۸. مخاطرات قابل پیش‌بینی کارآزمایی برای آزمودنی‌ها
۹. منافع موردانتظار برای شرکت‌کنندگان، چنان‌چه در یک کارآزمایی هیچ‌گونه منافعی پیش‌بینی نمی‌شودباید آزمودنی از آن آگاه باشد.
۱۰. در صورت استفاده از دارونما، توضیح معنای آن، احتمال تخصیص به گروه دارونما، و ذکر خطرات و فواید احتمالی در صورت تخصیص به شاخه یا گروه دریافت کننده‌ی دارونما
۱۱. روش‌های درمانی جایگزین که ممکن است دردسترس آزمودنی باشد به همراه منافع و خطرات بالقوه‌ی آن‌ها
۱۲. عدم تحمیل هزینه به آزمودنی به‌واسطه‌ی مداخلات پژوهشی
۱۳. غرامت و درمانصدماتی که در جریان کارآزمایی ممکن است برای فرد ایجاد شود.
۱۴. در صورتی‌کهوجهی در قبال مشارکت شرکت‌کنندگان در مطالعه پرداخت می‌شود،میزان و نحوه‌ی پرداخت آنذکر شود.
۱۵. بازپرداخت مخارجی که آزمودنی در اثر شرکت در مطالعه متحمل می‌شود.
۱۶. داوطلبانه بودن مشارکت افراد در کارآزمایی و تصریح به این‌که آزمودنی‌ها درهر مرحله از کارآزمایی این حق را دارند که از مطالعه خارج شوند بدون این‌که لازم باشد جریمه یا خسارتی را پرداخت کنند یا درمان معمول ایشان تحت تأثیر قرار گیرد.
۱۷. محرمانه‌بودن اطلاعات شخصی آزمودنی‌ها و تصریح به انتشار نتایج به‌صورت آماری و به‌نحوی که اطلاعات فردی فاش نشود.
۱۸. اشخاصی که حق دسترسی به اطلاعات آزمودنی را خواهند داشت، از جمله کمیته‌ی اخلاق در پژوهش
۱۹. تصریح به این‌که چنان‌چه اطلاعات جدیدی در موردسلامت افراد یا تأثیرگذار بر تداوم مشارکت آن‌ها در دسترس قرار گیرد، آزمودنی یا نماینده‌ی قانونی او در اولین فرصت در جریان قرار خواهند گرفت.
۲۰. نام و شماره‌ی تماس شخصیا اشخاصی که آزمودنی می‌تواند در زمان وقوع عوارض ناخواسته یا برای کسب اطلاعاتبیش‌تر با آن‌ها تماس بگیرد.
۲۱. پیش‌بینی و توصیف شرایطی که در آن شرایط، شرکت فرد در مطالعه ممکن است خاتمه یابد.
۲۲. مدت زمان مورد انتظار مشارکتافراد در کارآزمایی
۲۳. تعارض منافع احتمالی پژوهشگران و وابستگی‌هایحرفه‌ای ایشان

۱. چنان‌چه به هر دلیلی کارآزمایی قبل از موعدمقرر خاتمه یافته یا تعلیق شود، مؤسسه‌ی پژوهشی یا پژوهشگر باید آزمودنی‌را از اینموضوع مطلع کند و به او اطمینان دهد که درمان مناسب و پیگیری مورد نیاز برای آن‌ها انجام خواهد شد.

فصل سوم: دارونما

۱. فواید، خطرات، عوارض و کارآیی روشمورد آزمون باید در مقابل بهترین روش‌های پیش‌گیرانه، تشخیصی یا درمانی موجود موردمقایسه قرار گیرد.
۲. استفاده از دارونما در کارآزمایی‌های بالینی در صورتی که درمانیا مداخلات استاندارد وجود داشته باشد، غیرقابل قبول است، مگر در موارد ذیل

۲-۱ – شواهدی از اثربخشی بیش‌تر درمان استاندارد نسبت به دارونما وجود نداشته‌باشد

۲-۲ – درمان استاندارد به‌دلیل محدویت‌های هزینه یا عدم تأمینپایدار آن در دسترس نباشد. البته منظور از محدودیت‌های پرداخت هزینه از دیدگاه نظامسلامت است. بنابراین، این مورد شامل حالتی که تأمین درمان استاندارد اثربخشبرای افراد غنی یک جامعه ممکن و برای افراد کم‌درآمد غیرممکن باشد، نمی‌شود.

۲-۳ – چنان‌چه جامعه‌ی بیماران مورد مطالعه نسبت به درمان استاندارد مقاوم‌باشند و درمان استاندارد جایگزین برای آنان وجود نداشته باشد.

۲-۴ – وقتی‌که هدفکارآزمایی بررسی تأثیر توأم یک درمان به همراه درمان استاندارد باشد و به هر دلیلیکلیه‌ی افراد مورد مطالعه، درمان استاندارد را دریافت کرده باشند.

۲-۵ – وقتی کهبیماران درمان استاندارد را تحمل نمی‌کنند و اگر بیماران روی درمان استاندارد نگهداشته شوند، عوارض مرتبط با درمان و زیان‌های غیرقابل برگشت با هر شدتی برای آن‌ها ایجاد شود و درمان استاندارد جایگزینی برای آن‌ها وجود ندارد.

۲-۶ – زمانی‌که یک روش پیش‌گیری، تشخیص یا درمان برای یک وضعیت خفیفمورد بررسی قرار می‌گیرد و بیمارانی که دارونما دریافت می‌کنند در معرض خطر اضافی شدیدیا غیرقابل برگشتی قرار نمی‌گیرند.

۱. استفاده از جراحی دروغین به‌عنوان دارونما پذیرفته نیست مگر در مواردی‌که کلیه‌ی شروط زیر صادق باشد:

۳-۱- پیامد موردسنجش سوبژکتیو (ذهنی) باشد، از قبیل درد و کیفیت زندگی

۳-۲- جراحی استانداردقابل قیاس وجود نداشته باشد و تنها راه سنجش دقیق اثربخشی مداخله، استفاده ازکنترل جراحی دروغین باشد.

۳-۳- خطر جراحی دروغین به حد قابل قبولی پایینباشد.

۳-۴- بیمار با آزادی کامل و با آگاهی از این‌که ممکن است مورد جراحیدروغین قرار بگیرند که هیچ نفع درمانی برای آن‌ها ندارد، رضایت کتبی دادهباشد.

۳-۵- کمیته‌ی اخلاق انجام جراحی دروغین را در مورد مداخله مورد نظر بارعایت دستورالعمل ارائه شده مجاز تشخیص بدهد.

فصل چهارم: پرداخت غرامت

۱. هر گونه خسارت وارده به آزمودنی که ناشی از مشارکت او در کارآزمایی باشد، به‌نحوی که اگر فرد وارد مطالعه نمی‌شد چنین اتفاقی برای وی رخ نمی‌داد، باید به‌نحو مناسب جبران شود.
۲. در دستورالعمل کارآزمایی و فرم رضایت آگاهانه باید مشخص شود که مسؤولپرداخت غرامت چه فرد یا سازمانی است. در صورت مشخص نشدن این مورد، مجری اصلی کارآزمایی مسؤول جبران خسارت وارده و پرداخت غرامت است.
۳. جبران خسارت وارده به آزمودنی در کارآزمایی‌های بالینی در هر صورت باید جبران شود و مشروط به احراز تقصیر پژوهشگر نیست.
۴. موارد زیر مشمول پرداخت غرامت نمی‌شود:

۴-۱ – آسیب‌های جزیی مانند درد یا ناراحتی مختصر یا قابل درمان

۴-۲ – هنگامی که فرآورده یا داروی موردمطالعه نتواند اثر مورد انتظار را داشته باشد.

۴-۳ – در حین مصرف دارونما، بیماری رو به وخامت گذارد.

۴-۴ – آسیبی که در اثر تقصیر خود بیماررخ داده باشد.

۴-۵ – فاز ۴ کارآزمایی بالینی

۱. در مواردی که در مورد لزوم یا نحوه‌ی جبران خسارت، میان آزمودنی و پژوهشگر اختلاف نظر وجود داشته باشد، موضوع به کمیته‌ی اخلاق در پژوهش تأییدکننده‌ی مطالعه ارجاع شده و در کمیته تصمیم‌گیری می‌شود.

**محدودیت های اجرائی طرح و پیش بینی جهت حل آن ها:**	۹

عدم همکاری بیماران،ثبت اطلاعات اشتباه،مصرف کافئین بیشتر از مقدار توصیه شده و...

ث- جدول زمانبندی

الف- زمان طراحی پیش نویس طرح و تکمیل این فرم جزو زمان اجرای طرح محسوب نمی شود.
ب- دریافت گزارش های مرحله ای طرح با توجه به جدول گانت و طبق نظر دانشکده و استاد راهنما صورت می گیرد.
پ- پرداخت هزینه طرح براساس درخواست استاد راهنما و طبق مفاد قرارداد اجرای طرح صورت می گیرد
ت- زمان شروع طرح بعد از تصویب آن، با هماهنگی مجری/استاد راهنمای طرح های دانشجوئی و حوزه معاونت تحقیقات و فناوری می باشد.

ردیف	شرح مختصر مرحله	مدت اجرا
۱	نمونه گیری/انجام تست ها قبل از مصرف کافئین/انجام تست ها بعد از مصرف کافئین در هفته های۲،۴،۸،۱۲،۱۶،۲۰،۲۴/انجام مراحل آماری/تنظیم نتایج حاصل	۳۶۵

ج- اطلاعات مربوط به هزینه

هزینه های پرسنلی:

نوع فعالیت	کل ساعت	حق الزحمه هر ساعت (ریال)	جمع(ریال)
نظارت برانجام تست ها	۳۰.۰	۵۰۰۰۰	۱۵۰۰۰۰۰

وسایل و مواد مورد نیاز:

نام دستگاه/مواد	نوع وسیله	تعداد	قیمت به ریال	کل مبلغ
قرص کافئین	مصرفی	۲۰۲۵.۰	۵۰۰۰	۱۰۱۲۵۰۰۰

هزینه آزمایشات و خدمات تخصصی:

موضوع آزمایشات و یا خدمات تخصصی	نوع مرکز سرویس دهنده	نام مرکز سرویس دهنده	تعداد کل دفعات	هزینه برای هر دفعه یا تعرفه دولتی	جمع کل هزینه ها
بررسی تاثیر مصرف کافئین	دولتی	بیمارستا علی بن ابی طالب(ع) زاهدان	۸	۰	۰

هزینه مسافرت :

مقصد	نوع وسیله نقلیه	تعداد افراد	هدف از سفر	تعداد مسافرت در مدت اجرای طرح	هزینه هر سفر به ریال	مبلغ
بیمارستان علی بن ابی طالب(ع)	سواری	۳۱	انجام تست ها	۵۰	۶۰۰۰۰	۳۰۰۰۰۰۰

هزینه های متفرقه:

موضوع	سایر	هزینه (ریال)
هزینه تکثیر اوراق پرسشنامه		۲۰۰۰۰۰
هزینه تایپ و تکثیر طرح و گزارش نهایی		۱۷۵۰۰۰

تأمین بودجه از سایر منابع:

نام سازمان / اداره	مبلغ	مدت زمان

جمع هزینه های طرح:

جمع هزینه های پرسنلی	جمع هزینه های وسایل و مواد	جمع هزینه های آزمایشگاه و خدمات تخصصی	جمع هزینه های مسافرت	جمع هزینه های متفرقه	جمع کل هزینه ها
۱۵۰۰۰۰۰	۱۰۱۲۵۰۰۰	۰	۳۰۰۰۰۰۰	۳۷۵۰۰۰	۱۵۰۰۰۰۰۰

چ- جمع کل مبلغی که توسط معاونت تحقیقات و فناوری جهت اجرای طرح پیش بینی پرداخت آن شده است:

۱۵۰۰۰۰۰۰ ریال

ح- مبلغی که توسط منابع دیگر جهت اجرای طرح کمک خواهد شد:

۰ ریال

خ- منابع و مواخذ

۱. Gunn HJ, Newell P, Haas B, Marsden JF, Freeman JA. Identification of risk factors for falls in multiple sclerosis: a systematic review and meta-analysis. Physical therapy. ۲۰۱۳;۹۳(۴):۵۰۴-۱۳.

۲. Aharony S, Lam O, Lapierre Y, Corcos J. Multiple sclerosis (MS) for the urologist: What should urologists know about MS? Neurourology and urodynamics. ۲۰۱۶;۳۵(۲):۱۷۴-۹.

۳. Kalron A. The relationship between specific cognitive domains, fear of falling, and falls in people with multiple sclerosis. BioMed research international. ۲۰۱۴;۲۰۱۴.

۴. Van Asch P. Impact of mobility impairment in multiple sclerosis 2—patients' perspectives. Eur Neurol Rev. ۲۰۱۱;۶(۲):۱۱۵-۲۰.

۵. Souza A, Kelleher A, Cooper R, Cooper RA, Iezzoni LI, Collins DM. Multiple sclerosis and mobility-related assistive technology: systematic review of literature. Journal of rehabilitation research and development. ۲۰۱۰;۴۷(۳):۲۱۳.

۶. Martin CL, Phillips B, Kilpatrick T, Butzkueven H, Tubridy N, McDonald E, et al. Gait and balance impairment in early multiple sclerosis in the absence of clinical disability. Multiple sclerosis. ۲۰۰۶;۱۲(۵):۶۲۰-۸.

۷. Goodman AD, Brown TR, Edwards KR, Krupp LB, Schapiro RT, Cohen R, et al. A phase ۳ trial of extended release oral dalfampridine in multiple sclerosis. Annals of neurology. ۲۰۱۰;۶۸(۴):۴۹۴-۵۰۲.

۸. Goodman AD, Brown TR, Krupp LB, Schapiro RT, Schwid SR, Cohen R, et al. Sustained-release oral fampridine in multiple sclerosis: a randomised, double-blind, controlled trial. The Lancet. ۲۰۰۹;۳۷۳(۹۶۶۵):۷۳۲-۸.

۹. Fischer J, Rudick R, Cutter G, Reingold S. National MS Society Clinical Outcomes Assessment Task Force. The Multiple Sclerosis Functional Composite measure (MSFC): an integrated approach to MS clinical outcome assessment Mult Scler. ۱۹۹۹;۵(۴):۲۴۴-۵۰.

۱۰. LAC, dos Santos LT, Sabino PG, Alvarenga RMP, Santos Thuler Nogueira LC. Factors for lower walking speed in persons with multiple sclerosis. Multiple sclerosis international. ۲۰۱۳;۲۰۱۳.

۱۱. Nieuwenhuis M, Van Tongeren H, Sørensen P, Ravnborg M. The six spot step test: a new measurement for walking ability in multiple sclerosis. Multiple Sclerosis. ۲۰۰۶;۱۲(۴):۴۹۵-۵۰۰.

۱۲. Paltamaa J, Sarasoja T, Leskinen E, Wikström J, Mälkiä E. Measures of physical functioning predict self-reported performance in self-care, mobility, and domestic life in ambulatory persons with multiple sclerosis. Archives of physical medicine and rehabilitation. ۲۰۰۷;۸۸(۱۲):۱۶۴۹-۵۷.

۱۳. Horrigan LA, Kelly JP, Connor TJ. Immunomodulatory effects of caffeine: friend or foe? Pharmacology & therapeutics. ۲۰۰۶;۱۱۱(۳):۸۷۷-۹۲.

۱۴. Kalmar J, Cafarelli E. Effects of caffeine on neuromuscular function. Journal of applied physiology. ۱۹۹۹;۸۷(۲):۸۰۱-۸.

۱۵. Biaggioni I, Paul S, Puckett A, Arzubiaga C. Caffeine and theophylline as adenosine receptor antagonists in humans. Journal of Pharmacology and Experimental Therapeutics. ۱۹۹۱;۲۵۸(۲):۵۸۸-۹۳.

۱۶. Wentz CT, Magavi SS. Caffeine alters proliferation of neuronal precursors in the adult hippocampus. Neuropharmacology. ۲۰۰۹;۵۶(۶):۹۹۴-۱۰۰۰.

۱۷. Koppelstaetter F, Poeppel T, Siedentopf C, Ischebeck A, Verius M, Haala I, et al. Does caffeine modulate verbal working memory processes? An fMRI study. Neuroimage. ۲۰۰۸;۳۹(۱):۴۹۲-۹.

۱۸. Lieberman HR. Cognitive methods for assessing mental energy. Nutritional neuroscience. ۲۰۱۳.

۱۹. Walton C, Kalmar JM, Cafarelli E. Effect of caffeine on self-sustained firing in human motor units. The Journal of physiology. ۲۰۰۲;۵۴۵(۲):۶۷۱-۹.

۲۰. Hogervorst E, Bandelow S, Schmitt JA, Jentjens R, Oliveira M, Allgrove JE, et al. Caffeine improves physical and cognitive performance during exhaustive exercise. ۲۰۰۸.

۲۱. Addicott MA, Yang LL, Peiffer AM, Burnett LR, Burdette JH, Chen MY, et al. The effect of daily caffeine use on cerebral blood flow: How much caffeine can we tolerate? Human brain mapping. ۲۰۰۹;۳۰(۱۰):۳۱۰۲-۱۴.

۲۲. Philip P, Taillard J, Moore N, Delord S, Valtat C, Sagaspe P, et al. The effects of coffee and napping on nighttime highway driving: a randomized trial. Annals of internal medicine. ۲۰۰۶;۱۴۴(۱۱):۷۸۵-۹۱.

۲۳. Biggs SN, Smith A, Dorrian J, Reid K, Dawson D, Van den Heuvel C, et al. Perception of simulated driving performance after sleep restriction and caffeine. Journal of psychosomatic research. ۲۰۰۷;۶۳(۶):۵۷۳-۷.

۲۴. Peeling P, Dawson B. Influence of caffeine ingestion on perceived mood states, concentration, and arousal levels during a ۷۵-min university lecture. Advances in physiology education. ۲۰۰۷;۳۱(۴):۳۳۲-۵.

۲۵. Kennedy MD, Galloway AV, Dickau LJ, Hudson MK. The cumulative effect on heart rate, blood pressure, and mental of coffee and a mental stress task alertness is similar in caffeine-naïve and caffeine-habituated females. Nutrition research. ۲۰۰۸;۲۸(۹):۶۰۹-۱۴.

۲۶. Evans SM, Griffiths RR. Caffeine tolerance and choice in humans. Psychopharmacology. ۱۹۹۲;۱۰۸(۱-۲):۹-۵۱.

۲۷. Hadjicharalambous M, Georgiades E, Kilduff LP, Turner A, Tsofliou F, Pitsiladis Y. Influence of caffeine on perception of effort, metabolism and exercise performance following a high-fat meal. Journal of sports sciences. ۲۰۰۶;۲۴(۸):۸۷۵-۸۷.

۲۸. Hamer M. Coffee and health: explaining conflicting results in hypertension. Journal of human hypertension. ۲۰۰۶;۲۰(۱۲):۹۰۹-۱۲.

۲۹. Lopez-Garcia E, van Dam RM, Li TY, Rodriguez-Artalejo F, Hu FB. The relationship of coffee consumption with mortality. Annals of internal medicine. ۲۰۰۸;۱۴۸(۱۲):۹۰۴-۱۴.

۳۰. Maas A, Hogenhuis L. Multiple sclerosis and possible relationship to cocoa: a hypothesis. Annals of allergy. ۱۹۸۷;۵۹(۱):۷۶.

۳۱. Evans SM, Griffiths RR. Caffeine withdrawal: a parametric analysis of dosing conditions. Journal of Pharmacology and Experimental caffeine Therapeutics. ۱۹۹۹;۲۸۹(۱):۲۸۵-۹۴.

۳۲. Miri S, Rohani M, Sahraian MA, Zamani B, Shahidi GA, Sabet A, et al. Restless legs syndrome in Iranian patients with multiple sclerosis. Neurological Sciences. ۲۰۱۳;۳۴(۷):۱۱۰۵-۸.

۳۳. Massa J, O'Reilly E, Munger K, Ascherio A. Caffeine and alcohol intakes have no association with risk of multiple sclerosis. Multiple Sclerosis Journal. ۲۰۱۳;۱۹(۱):۵۳-۸.

۳۴. Hedström A, Mowry EM, Gianfrancesco M, Shao X, Schaefer C, Shen L, et al. High consumption of coffee is associated with decreased multiple sclerosis risk; results from two independent studies. Journal of Neurology, Neurosurgery & Psychiatry. ۲۰۱۶:jnnp-۲۰۱۵-۳۱۲۱۷۶.

Chen J-f, et al. Chronic caffeine ,Wang T, Xi N-n, Chen Y, Shang X-f, Hu Q .۳۵ treatment protects against experimental autoimmune encephalomyelitis in mice: Therapeutic window and receptor subtype mechanism. .Neuropharmacology. ۲۰۱۴;۸۶:۲۰۳-۱۱

Kiernan MC, et al. ,Pickering H, Murray J, Lin CS-Y, Cormack C, Martin A .۳۶ Fampridine treatment and walking distance in multiple sclerosis: A .randomised controlled trial. Clinical Neurophysiology. ۲۰۱۷;۱۲۸(۱):۹۳-۹

Brambilla L, Sebastiano DR, Aquino D, Clerici VT, Brenna G, Moscatelli M, .۳۷ et al. Early effect of dalfampridine in patients with MS: A multi-instrumental approach to better investigate responsiveness. Journal of the Neurological .Sciences. ۲۰۱۶;۳۶۸:۴۰۲-۷

د – ضمائم

پرسشنامه

Kurtzke Expanded Disability Status Scale (EDSS)

❑ ۰.۰ – Normal neurological exam (all grade ۰ in all Functional System (FS) scores*).

❑ ۱.۰ – No disability, minimal signs in one FS* (i.e., grade ۱).

❑ ۱.۵ – No disability, minimal signs in more than one FS* (more than ۱ FS grade ۱).

❑ ۲.۰ – Minimal disability in one FS (one FS grade ۲, others ۰ or ۱).

❑ ۲.۵ – Minimal disability in two FS (two FS grade ۲, others ۰ or ۱).

❑ ۳.۰ – Moderate disability in one FS (one FS grade ۳, others ۰ or ۱) or mild disability in three or four FS (three or four FS grade ۲, others ۰ or ۱) though fully ambulatory.

❑ ۳.۵ – Fully ambulatory but with moderate disability in one FS (one grade ۳) and one or two FS grade ۲; or two FS grade ۳ (others ۰ or ۱) or five grade ۲ (others ۰ or ۱).

❑ ۴.۰ – Fully ambulatory without aid, self-sufficient, up and about some ۱۲ hours a day despite relatively severe disability consisting of one FS grade ۴ (others ۰ or ۱), or combination of lesser grades exceeding limits of previous steps; able to walk without aid or rest some ۵۰۰ meters.

❑ ۴.۵ – Fully ambulatory without aid, up and about much of the day, able to work a full day, may otherwise have some limitation of full activity or require minimal assistance; characterized by relatively severe disability usually consisting of one FS grade ۴ (others or ۱) or combinations of lesser grades exceeding limits of previous steps; able to walk without aid or rest some ۳۰۰ meters.

❑ ۵.۰ – Ambulatory without aid or rest for about ۲۰۰ meters; disability severe enough to impair full daily activities (e.g., to work a full day without special

provisions); (Usual FS equivalents are one grade ۵ alone, others ۰ or ۱; or combinations of lesser grades usually exceeding specifications for step ۴.۰).

❑ ۵.۵ – Ambulatory without aid for about ۱۰۰ meters; disability severe enough to preclude full daily activities; (Usual FS equivalents are one grade ۵ alone, others ۰ or ۱; or combination of lesser grades usually exceeding those for step ۴.۰).

❑ ۶.۰ – Intermittent or unilateral constant assistance (cane, crutch, brace) required to walk about ۱۰۰ meters with or without resting; (Usual FS equivalents are combinations with more than two FS grade ۳+).

❑ ۶.۵ – Constant bilateral assistance (canes, crutches, braces) required to walk about ۲۰ meters without resting; (Usual FS equivalents are combinations with more than two FS grade ۳+).

❑ ۷.۰ – Unable to walk beyond approximately ۵ meters even with aid, essentially restricted to wheelchair; wheels self in standard wheelchair and transfers alone; up and about in wheelchair some ۱۲ hours a day; (Usual FS equivalents are combinations with more than one FS grade ۴+; very rarely pyramidal grade ۵ alone).

❑ ۷.۵ – Unable to take more than a few steps; restricted to wheelchair; may need aid in transfer; wheels self but cannot carry on in standard wheelchair a full day; May require motorized wheelchair; (Usual FS equivalents are combinations with more than one FS grade ۴+).

❑ ۸.۰ – Essentially restricted to bed or chair or perambulated in wheelchair, but may be out of bed itself much of the day; retains many self-care functions; generally has effective use of arms; (Usual FS equivalents are combinations, generally grade ۴+ in several systems).

❑ ۸.۵ – Essentially restricted to bed much of day; has some effective use of arm(s); retains some self-care functions; (Usual FS equivalents are combinations, generally ۴+ in several systems).

❑ ۹.۰ – Helpless bed patient; can communicate and eat; (Usual FS equivalents are combinations, mostly grade ۴+).

❑ ۹.۵ – Totally helpless bed patient; unable to communicate effectively or eat/swallow; (Usual FS equivalents are combinations, almost all grade ۴+). ❑ ۱۰.۰ – Death due to MS.

*Excludes cerebral function grade ۱.

Note ۱: EDSS steps ۱.۰ to ۴.۵ refer to patients who are fully ambulatory and the precise step number is defined by the Functional System score(s). EDSS steps ۵.۰ to ۹.۵ are defined by the impairment to ambulation and usual equivalents in Functional Systems scores are provided.

Note ۲: EDSS should not change by ۱.۰ step unless there is a change in the same direction of at least one step in at least one FS.

Twelve Item MS Walking Scale (MSWS-۱۲)

In the past two weeks, how much has your MS . . .	Not at all	A little	Moderately	Quite a lot	Extremely
۱. Limited your ability to walk?	۱	۲	۳	۴	۵
۲. Limited your ability to run?					
۳. Limited your ability to climb up and down stairs?					
۴. Made standing when doing things more difficult?					
۵. Limited your balance when standing or walking?					

۶. Limited how far you are able to walk?					
۷. Increased the effort needed for you to walk?					
۸. Made it necessary for you to use support when walking indoors (eg holding on to furniture, using a stick, etc.)?					
۹. Made it necessary for you to use support when walking outdoors (eg using a stick, a frame, etc.)?					
۱۰. Slowed down your walking?					
۱۱. Affected how smoothly you walk?					
۱۲. Made you concentrate on your walking?					

From the numbers you circle against these questions, your healthcare professional can calculate your MSWS-۱۲ score. This is done by adding the

numbers you have circled, giving a total out of ۶۰, and then transforming this to a scale with a range from ۰ to ۱۰۰. Higher scores indicate a greater impact on walking than lower scores.

To be completed by the healthcare professional

Total score ..... out of ۶۰

Percentage...... ٪

Patients' Global Impression of Change (PGIC) scale

Name:...... Date:....... DOB:...... Chief Complaint......

Since beginning treatment at this clinic how would you describe the change (if any)in activity limitations,symptoms,emothions and overall quality of life related to your painful condition? (thick one box)

۱-no change or condition has got worse

۲-almost the same hardly any change at all

۳-a little change but no noticeable changes

۴-somewhat better but the change has not made any real difference

۵-moderatly better and a slight but noticeable change

۶-better and a definite improvement that has made a real and worthwhile difference

۷-a great deal better and a considerable improvement that has made all the difference

Patient's signature:...... Date:......

The Timed Up and Go (TUG) Test

Purpose: To assess mobility

Equipment: A stopwatch

Directions: Patients wear their regular footwear and can use a walking aid if needed. Begin by having the patient sit back in a standard arm chair and identify a line ۳ meters or ۱۰ feet away on the floor.

Instructions to the patient: When I say “Go,” I want you to:

۱. Stand up from the chair

۲. Walk to the line on the floor at your normal pace

۳. Turn

۴. Walk back to the chair at your normal pace

۵. Sit down again

On the word "Go" begin timing.

Stop timing after patient has sat back down and record.

Time: __________ seconds

An older adult who takes ≥۱۲ seconds to complete the TUG is at high risk for falling.

Observe the patient's postural stability, gait, stride length, and sway.

Circle all that apply: Slow tentative pace n Loss of balance n Short strides n Little or no arm swing n Steadying self on walls n Shuffling n En bloc turning n Not using assistive device properly

Notes:......

Berg Balance Scale

The Berg Balance Scale (BBS) was developed to measure balance among older people with impairment in balance function by assessing the performance of functional tasks. It is a valid instrument used for evaluation of the effectiveness of interventions and for quantitative descriptions of function in clinical practice and research. The BBS has been evaluated in several reliability studies. A recent study of the BBS, which was completed in Finland, indicates that a change of eight (۸) BBS points is required to reveal a genuine change in function between two assessments among older people who are dependent in ADL and living in residential care facilities.

Description: ۱۴-item scale designed to measure balance of the older adult in a clinical setting.

Equipment needed: Ruler, two standard chairs (one with arm rests, one without), footstool or step, stopwatch or wristwatch, ۱۵ ft walkway

Completion: Time: ۱۵-۲۰ minutes.

Scoring: A five-point scale, ranging from ۰-۴. "۰" indicates the lowest level of function and "۴" the highest level of function. Total Score = ۵۶

Interpretation:

۴۱-۵۶ = low fall risk

۲۱-۴۰ = medium fall risk

۰ –۲۰ = high fall risk

A change of ۸ points is required to reveal a genuine change in function between ۲ assessments.

Berg Balance Scale Name: ______________________________________ Date: _____________________ Location: ____________________________________ Rater: _____________________ ITEM DESCRIPTION SCORE (۰-۴)

Sitting to standing _________

Standing unsupported _________

Sitting unsupported _________

Standing to sitting _________

Transfers _________

Standing with eyes closed _________

Standing with feet together _________

Reaching forward with outstretched arm _________

Retrieving object from floor _________

Turning to look behind _________

Turning ۳۶۰ degrees _________

Placing alternate foot on stool _________

Standing with one foot in front _________

Standing on one foot _________

Total _________

GENERAL INSTRUCTIONS

Please document each task and/or give instructions as written. When scoring, please record the lowest response category that applies for each item.

In most items, the subject is asked to maintain a given position for a specific time. Progressively more points are deducted if:

- the time or distance requirements are not met
- the subject's performance warrants supervision
- the subject touches an external support or receives assistance from the examiner

Subject should understand that they must maintain their balance while attempting the tasks. The choices of which leg to stand on or how far to reach are left to the subject. Poor judgment will adversely influence the performance and the scoring.

Equipment required for testing is a stopwatch or watch with a second hand, and a ruler or other indicator of ۲, ۵, and ۱۰ inches. Chairs used during testing should be a reasonable height. Either a step or a stool of average step height may be used for item # ۱۲.

-SITTING TO STANDING

INSTRUCTIONS: Please stand up. Try not to use your hand for support.

( ) ۴ able to stand without using hands and stabilize independently

( ) ۳ able to stand independently using hands

( ) ۲ able to stand using hands after several tries

( ) ۱ needs minimal aid to stand or stabilize

( ) ۰ needs moderate or maximal assist to stand STANDING UNSUPPORTED INSTRUCTIONS:

Please stand for two minutes without holding on.

( ) ۴ able to stand safely for ۲ minutes

( ) ۳ able to stand ۲ minutes with supervision

( ) ۲ able to stand ۳۰ seconds unsupported

( ) ۱ needs several tries to stand ۳۰ seconds unsupported

( ) ۰ unable to stand ۳۰ seconds unsupported

If a subject is able to stand ۲ minutes unsupported, score full points for sitting unsupported. Proceed to item #۴.

SITTING WITH BACK UNSUPPORTED BUT FEET SUPPORTED ON FLOOR OR ON A STOOL INSTRUCTIONS:

Please sit with arms folded for ۲ minutes.

( ) ۴ able to sit safely and securely for ۲ minutes

( ) ۳ able to sit ۲ minutes under supervision

( ) ۲ able to able to sit ۳۰ seconds

( ) ۱ able to sit ۱۰ seconds

( ) ۰ unable to sit without support ۱۰ seconds

STANDING TO SITTING INSTRUCTIONS:

Please sit down.

( ) ۴ sits safely with minimal use of hands

( ) ٣ controls descent by using hands

( ) ٢ uses back of legs against chair to control descent

( ) ١ sits independently but has uncontrolled descent

( ) ٠ needs assist to sit

TRANSFERS INSTRUCTIONS:

Arrange chair(s) for pivot transfer. Ask subject to transfer one way toward a seat with armrests and one way toward a seat without armrests. You may use two chairs (one with and one without armrests) or a bed and a chair.

( ) ۴ able to transfer safely with minor use of hands

( ) ٣ able to transfer safely definite need of hands

( ) ٢ able to transfer with verbal cuing and/or supervision

( ) ١ needs one person to assist

( ) ٠ needs two people to assist or supervise to be safe STANDING UNSUPPORTED WITH EYES CLOSED INSTRUCTIONS:

Please close your eyes and stand still for ١٠ seconds.

( ) ۴ able to stand ١٠ seconds safely

( ) ٣ able to stand ١٠ seconds with supervision

( ) ٢ able to stand ٣ seconds

( ) ١ unable to keep eyes closed ٣ seconds but stays safely

( ) ٠ needs help to keep from falling

STANDING UNSUPPORTED WITH FEET TOGETHER INSTRUCTIONS:

Place your feet together and stand without holding on.

( ) ۴ able to place feet together independently and stand ١ minute safely

( ) ٣ able to place feet together independently and stand ١ minute with supervision

( ) ٢ able to place feet together independently but unable to hold for ٣٠ seconds

( ) ١ needs help to attain position but able to stand ١۵ seconds feet together

( ) ٠ needs help to attain position and unable to hold for ١۵ seconds Berg Balance Scale continued...

REACHING FORWARD WITH OUTSTRETCHED ARM WHILE STANDING INSTRUCTIONS:

Lift arm to ٩٠ degrees. Stretch out your fingers and reach forward as far as you can. (Examiner places a ruler at the end of fingertips when arm is at ٩٠ degrees. Fingers should not touch the ruler while reaching forward. The

recorded measure is the distance forward that the fingers reach while the subject is in the most forward lean position. When possible, ask subject to use both arms when reaching to avoid rotation of the trunk.)

( ) ۴ can reach forward confidently ۲۵ cm (۱۰ inches)

( ) ۳ can reach forward ۱۲ cm (۵ inches)

( ) ۲ can reach forward ۵ cm (۲ inches)

( ) ۱ reaches forward but needs supervision

( ) ۰ loses balance while trying/requires external support

PICK UP OBJECT FROM THE FLOOR FROM A STANDING POSITION INSTRUCTIONS:

Pick up the shoe/slipper, which is in front of your feet.

( ) ۴ able to pick up slipper safely and easily

( ) ۳ able to pick up slipper but needs supervision

( ) ۲ unable to pick up but reaches ۲-۵ cm(۱-۲ inches) from slipper and keeps balance independently

( ) ۱ unable to pick up and needs supervision while trying

( ) ۰ unable to try/needs assist to keep from losing balance or falling TURNING TO LOOK BEHIND OVER LEFT AND RIGHT SHOULDERS WHILE STANDING INSTRUCTIONS:

Turn to look directly behind you over toward the left shoulder. Repeat to the right. (Examiner may pick an object to look at directly behind the subject to encourage a better twist turn.)

( ) ۴ looks behind from both sides and weight shifts well

( ) ۳ looks behind one side only other side shows less weight shift

( ) ۲ turns sideways only but maintains balance

( ) ۱ needs supervision when turning ( ) ۰ needs assist to keep from losing balance or falling TURN ۳۶۰ DEGREES INSTRUCTIONS:

Turn completely around in a full circle. Pause. Then turn a full circle in the other direction.

( ) ۴ able to turn ۳۶۰ degrees safely in ۴ seconds or less

( ) ۳ able to turn ۳۶۰ degrees safely one side only ۴ seconds or less

( ) ۲ able to turn ۳۶۰ degrees safely but slowly

( ) ۱ needs close supervision or verbal cuing

( ) ۰ needs assistance while turning

PLACE ALTERNATE FOOT ON STEP OR STOOL WHILE STANDING UNSUPPORTED INSTRUCTIONS:

Place each foot alternately on the step/stool. Continue until each foot has touched the step/stool four times.

( ) ۴ able to stand independently and safely and complete ۸ steps in ۲۰ seconds

( ) ۳ able to stand independently and complete ۸ steps in > ۲۰ seconds

( ) ۲ able to complete ۴ steps without aid with supervision

( ) ۱ able to complete > ۲ steps needs minimal assist

( ) ۰ needs assistance to keep from falling/unable to try

STANDING UNSUPPORTED ONE FOOT IN FRONT INSTRUCTIONS: (DEMONSTRATE TO SUBJECT) Place one foot directly in front of the other. If you feel that you cannot place your foot directly in front, try to step far enough ahead that the heel of your forward foot is ahead of the toes of the other foot. (To score ۳ points, the length of the step should exceed the length of the other foot and the width of the stance should approximate the subject's normal stride width.)

( ) ۴ able to place foot tandem independently and hold ۳۰ seconds

( ) ۳ able to place foot ahead independently and hold ۳۰ seconds

( ) ۲ able to take small step independently and hold ۳۰ seconds

( ) ۱ needs help to step but can hold ۱۵ seconds

( ) ۰ loses balance while stepping or standing

STANDING ON ONE LEG INSTRUCTIONS:

Stand on one leg as long as you can without holding on.

( ) ۴ able to lift leg independently and hold > ۱۰ seconds

( ) ۳ able to lift leg independently and hold ۵-۱۰ seconds

( ) ۲ able to lift leg independently and hold ≥ ۳ seconds

( ) ۱ tries to lift leg unable to hold ۳ seconds but remains standing independently.

( ) ۰ unable to try of needs assist to prevent fall

( ) TOTAL SCORE (Maximum = ۵۶)

Multiple Sclerosis Impact Scale (MSIS-۲۹)

The following questions ask for your views about the impact of MS on your day-to-day life **during the past two weeks**

- For each statement, please **circle** the **one** number that **best** describes your situation

- Please answer **all** questions

	**In the past two weeks, how much has your MS limited your ability to...**	Not at all	A little	Moderately	Quite a bit	Extremely
۱.	**Do physically demanding tasks?**	۱	۲	۳	۴	۵
۲.	**Grip things tightly (e.g. turning on taps)?**	۱	۲	۳	۴	۵
۳.	**Carry things?**	۱	۲	۳	۴	۵

	**In the past two weeks, how much have you been bothered by...**	Not at all	A little	Moderately	Quite a bit	Extremely
۴.	**Problems with your balance?**	۱	۲	۳	۴	۵

۵.	**Difficulties moving about indoors?**	۱	۲	۳	۴	۵
۶.	**Being clumsy?**	۱	۲	۳	۴	۵
۷.	**Stiffness?**	۱	۲	۳	۴	۵
۸.	**Heavy arms and/or legs?**	۱	۲	۳	۴	۵
۹.	**Tremor of your arms or legs?**	۱	۲	۳	۴	۵
۱۰.	**Spasms in your limbs?**	۱	۲	۳	۴	۵
۱۱.	**Your body not doing what you want it to do?**	۱	۲	۳	۴	۵
۱۲.	**Having to depend on others to do things for you?**	۱	۲	۳	۴	۵

Please check that you have answered all the questions before going on to the next page

®۲۰۰۰ Neurological Outcome Measures Unit, ۴th Floor Queen Mary Wing, NHNN, Queen Square, London WC۱N ۳BG, UK

	**In the <u>past two weeks</u>, how much have you been bothered by…**	Not at all	A little	Moderately	Quite a bit	Extremely
۱۳.	**Limitations in your social and leisure activities at home?**	۱	۲	۳	۴	۵
۱۴.	**Being stuck at home more than you would like to be?**	۱	۲	۳	۴	۵
۱۵.	**Difficulties using your hands in everyday tasks?**	۱	۲	۳	۴	۵
۱۶.	**Having to cut down the amount of time you spent on work or other daily activities?**	۱	۲	۳	۴	۵
۱۷.	**Problems using transport (e.g. car, bus, train, taxi, etc.)?**	۱	۲	۳	۴	۵

۱۸.	**Taking longer to do things?**	۱	۲	۳	۴	۵
۱۹.	**Difficulty doing things spontaneously (e.g. going out on the spur of the moment)?**	۱	۲	۳	۴	۵
۲۰.	**Needing to go to the toilet urgently?**	۱	۲	۳	۴	۵
۲۱.	**Feeling unwell?**	۱	۲	۳	۴	۵
۲۲.	**Problems sleeping?**	۱	۲	۳	۴	۵
۲۳.	**Feeling mentally fatigued?**	۱	۲	۳	۴	۵
۲۴.	**Worries related to your MS?**	۱	۲	۳	۴	۵
۲۵.	**Feeling anxious or tense?**	۱	۲	۳	۴	۵
۲۶.	**Feeling irritable, impatient, or short tempered?**	۱	۲	۳	۴	۵

۲۷.	**Problems concentrating?**	۱	۲	۳	۴	۵
۲۸	**Lack of confidence?**	۱	۲	۳	۴	۵
۲۹.	**Feeling depressed?**	۱	۲	۳	۴	۵

Please check that you have circled ONE number for EACH question

Ó ۲۰۰۰ Neurological Outcome Measures Unit, ۴th Floor Queen Mary Wing, NHNN, Queen Square, London WC۱N ۳BG, UK

رضایت نامه آگاهانه

آیتم رضایت نامه	متن
معرفی پژوهش	**رضایت نامه شرکت در طرح** بررسی تاثیر مصرف کافئین بر راه رفتن و تعادل در بیماران مبتلا به مالتیپل اسکلروزیس در بیمارستان علی بن ابی طالب(ع) در سال ۱۳۹۵<br><br>**آقای/ خانم محترم**<br><br>بدین وسیله از شما جهت شرکت در پژوهش فوق‌الذکر دعوت به عمل می‌آید. اطلاعات مربوط به این پژوهش در این برگه خدمتتان ارائه شده است و شما برای شرکت یا عدم شرکت در این پژوهش آزاد هستید.

	شما مجبور به تصمیم گیری فوری نیستید و برای تصمیم گیری در این باره می‌توانید سوالات خود را از تیم پژوهشی بپرسید و با هر فردی که مایل باشید مشورت نمایید. قبل از امضای این رضایت نامه مطمئن شوید که متوجه تمامی اطلاعات این فرم شده‌اید و به تمام سوالات شما پاسخ داده شده است.<br><br>**مجری پژوهش:افسون دادور**
خون گیری	
مزایا	۱.منافع احتمالی شرکت اینجانب در این مطالعه به این شرح است:<br><br>در این طرح هیچ سود مستقیمی برای بیمار در نظر گرفته نشده است و شرکت آنها در پژوهش میتواند به بهبود روشهای تشخیصی و درمانی بیماران آینده کمک کند را ذکر کنید.
عوارض جانبی	آسیب‌ها و عوارض احتمالی شرکت در این مطالعه به این شرح است:ممکن است بیمار دچار تپش قلب،پرادراری و یا بی خوابی گردد.
جبران عوارض احتمالی	در صورت دیده شدن هرکدام از عوارض احتمالی دوز دارو کمتر شده و در صورت واکنش شدید آزمایشات برای آن بیمار دیگر انجام نخواهد گرفت و بیمار به صورت رایگان درمان خواهد شد<br><br>من می‌دانم که اگر در حین و بعد از انجام پژوهش هر مشکلی اعم از جسمی و روحی به علت شرکت در این پژوهش برای من پیش آمد درمان عوارض، و هزینه‌های آن و غرامت مربوطه بر عهده مجری خواهد بود. .
هزینه های مربوط به انجام طرح تحقیقاتی	من می‌دانم که هیچ‌یک از هزینه‌های انجام مداخلات پژوهشی به شرح ذیل بر عهده من نخواهد بود.<br><br>هزینه قرص های کافئین و انجام تست ها به هیچ وجه برای بیمار هزینه ای نخواهد داشت.<br><br>من می‌دانم که اگر در حین و بعد از انجام پژوهش هر مشکلی اعم از جسمی و روحی به علت شرکت در این پژوهش برای من پیش آمد درمان عوارض، و هزینه‌های آن و غرامت مربوطه بر عهده مجری خواهد بود.
روش های جایگزین	
محرمانه بودن	۱. من میدانم که دست اندر کاران این پژوهش، کلیه اطلاعات مربوط به من را نزد خود به صورت محرمانه نگه‌داشته و فقط اجازه دارند فقط نتایج کلی و گروهی این پژوهش را بدون ذکر نام و مشخصات اینجانب منتشر کنند.<br>۲. میدانم که کمیته اخلاق در پژوهش با هدف نظارت بر رعایت حقوق اینجانب می‌تواند به اطلاعات من دسترسی داشته باشد.
پاسخگویی به پرسش ها	۱. خانم افسون دادور جهت پاسخگویی به اینجانب معرفی شد و به من گفته شد تا هر وقت مشکلی یا سوالی در رابطه با شرکت در پژوهش مذکور پیش آمد با ایشان در میان بگذارم و راهنمایی بخواهم.آدرس و شماره تلفن ثابت و همراه ایشان به شرح به من ارائه شد:

	**دانشکده علوم پزشکی زاهدان-۰۹۰۱۷۴۳۲۵۲۹آدرس:**<br>**میدان دکترحسابی**
حق نپذیرفتن یا انصراف	من میدانم که شرکت من در این پژوهش کاملاً داوطلبانه است و مجبور به شرکت در این پژوهش نیستم.<br>به من اطمینان داده شد که اگر حاضر به شرکت در این پژوهش نباشم، از مراقبت‌های معمول تشخیصی و درمانی محروم نخواهم شد و رابطه درمانی من با مرکز درمانی و پزشک معالجم دچار اشکال نمی‌شود.<br>۱. من می‌دانم که حتی پس از موافقت با شرکت در پژوهش می‌توانم هر وقت که بخواهم، پس از اطلاع به مجری، از پژوهش خارج شوم و خروج من از پژوهش باعث محرومیت از دریافت خدمات درمانی معمول برای من نخواهد شد.
رضایت	اینجانب موارد فوق‌الذکر را خواندم و فهمیدم و بر اساس آن رضایت آگاهانه خود را برای شرکت در این پژوهش اعلام می‌کنم.<br>امضای شرکت کننده<br>اینجانب .... خود را ملزم به اجرای تعهدات مربوط به مجری در مفاد فوق دانسته و متعهد می‌گردم در تأمین حقوق و ایمنی شرکت کننده در این پژوهش تلاش نمایم.<br>مهر و امضای مجری پژوهش

کاربست

کادر ۱ : مخاطب/مخاطبین

ردیف	گروه مخاطب
