# Supplementary material for: Potential efficacy of caffeine ingestion on balance and mobility in patients with multiple sclerosis: Preliminary evidence from a single-arm pilot clinical trial
Source: PLoS One. 2024 Feb 13;19(2):e0297235. doi: 10.1371/journal.pone.0297235 (PMC10863863; doi:10.1371/journal.pone.0297235)
Supplement: S3 File — (PDF) [file pone.0297235.s003.pdf]

وزارت بهداشت، درمان و آموزش پزشکی

دانشگاه علوم پزشکی زاهدان

معاونت تحقیقات و فناوری

Ministry of Health and Medical Education

Zahedan University of Medical Sciences

Deputy of Research and Technology

Title:

The effects of caffeine on walking and balance in patients with multiple sclerosis at Ali ibn Abi Talib Hospital

Study ID:

8029

The date of approval in the faculty research council:

October 25, 2016

The date of approval in the ethics committee of the university:

December 18, 2016

The date of approval in the university research council:

April 7, 2017

Keywords:

Caffeine; Balance; Multiple Sclerosis

Statement of the problem:

Multiple sclerosis (MS) is the most common cause of disability in young people. The prevalence of this disease in the world today has increased, so that about 2.5 million people in the world are suffering from this disease (1). MS can cause fatigue, sensory disorders, pain, deficits in mobility and balance, cognitive impairment, and visual symptoms (1). Studies have shown that genetics and environmental factors are among the MS predisposing factors (2). Due to cognitive disorders, patients with MS (PwMS) face problems in processing speed, attention, executive function, learning, and memory (3). Inappropriate and weak postural control, disability, fear of falling, walking problems, sensory problems, and the use of assistive devices are among the most important problems in PwMS (3). Movement disorder is one of the common aspects of this disease (4, 5), and sometimes it is the first symptom (6). Fampridine is the only medication that is used to improve walking speed in PwMS (7-9). Walking is a complex process that is affected by many factors, including balance disturbance (10,11), which is frequently seen among PwMS (12), resulting in reduced walking speed (10).

Caffeine is an alkaloid with the formula $C_8H_{10}N_4O_2$, which is found in beverages such as tea, coffee, cola, carbonated soft drinks, and energy drinks (13-15). Caffeine stimulates the central nervous system in humans (14, 16). Its effects include improving the neural transmission in the sympathetic nervous system (17), increasing the basal metabolic rate (18), improving motor function (19), cognitive function (20), mental energy (18,21), and neuromuscular coordination (22,23). Additionally, caffeine causes an increase in the serotonin concentration in the brain stem, resulting in the activation of skeletal motor units through motor neuron stimulation (19). Furthermore, consuming 32-50 mg of caffeine considerably improves arousal levels and the ability to concentrate in less than 20 minutes (24). Consuming 140 mg of caffeine resulted in a significant increase in the mental alertness of people, even after 50 minutes (25). The amount of caffeine required for its stimulating effects depends on the body mass index and the level of people's tolerance, and the amount of consumption is different for different people. Consumption of caffeine up to 900 mg per day is not dangerous for health, but it should not be consumed at once. Instead, it should be divided into three

doses of 300 mg per day (26). Usually, the amount of this substance increases in the blood within 15-45 minutes and reaches its maximum blood concentration within an hour, with a half-life of nearly 5 hours (17,27). Retrospective studies have suggested a relationship between the consumption of 3 or more cups of coffee per day with an increase in cardiovascular disease, but prospective studies have not confirmed any relationship between the consumption of any amount of caffeine and cardiovascular disease (28,29).

Possible adverse events of caffeine include insomnia, diuresis, and aggravation of migraine symptoms or digestive disorders (30). With respect to the concerns regarding caffeine addiction, previous studies have shown that only in the case of a sudden reduction of more than 100 mg per day is there a possibility of caffeine withdrawal symptoms (31). Additionally, no relationship has been found between caffeinated drinks and restless leg syndrome (32). Previous studies have suggested that caffeine consumption not only does not increase the risk of MS disease (33) but also might reduce disease susceptibility or progression among people who consume a large amount of coffee (900 milliliters per day) (34). Previous animal studies have also shown that due to the fact that caffeine is an adenosine receptor antagonist, it can prevent encephalomyelitis caused by the autoimmune disease and reduces brain damage in MS (35).

In light of these findings and also since there is no approved medication to improve walking in PwMS, except for fampridine which is only effective in a subset of PwMS and may also cause serious side effects, this is the first study aimed to investigate the effect of caffeine on the ambulatory performance of PwMS.

Research questions and/or hypotheses:

- Does caffeine consumption improve ambulatory performance in PwMS?
- Does caffeine consumption improve the objective assessment of static and dynamic balance in PwMS? (Assessed using the Berg Balance Scale [BBS])
- Does caffeine consumption improve the objective assessment of dynamic balance and functional mobility in PwMS? (Assessed using the Timed Up-and-Go [TUG])
- Does caffeine consumption improve the subjective assessment of walking abilities in PwMS? (Assessed using the 12-item Multiple Sclerosis Walking Scale [MSWS-12])
- Does caffeine consumption improve the subjective assessment of the impact of MS on the patient's quality of life in PwMS? (Assessed using the Multiple Sclerosis Impact Scale [MSIS-29])
- Does caffeine consumption improve subjective assessment of treatment efficacy in PwMS? (Assessed using the Patient's Global Impression of Change [PGIC])

Independent variables and outcome measures:

Age	Independent	Quantitative	Years<br>18-65
Sex	Independent	Qualitative	Male, Female
Comorbidities	Independent	Qualitative	Any other comorbidity
Habitual History	Independent	Qualitative	Yes, No<br>(Smoking, Alcohol, Hookah)
Education	Independent	Quantitative	Years
Job	Independent	Qualitative	Unemployed<br>Employed<br>Retired
MS phenotype	Independent	Qualitative	RRMS<br>SPMS<br>PPMS
Age of disease onset	Independent	Quantitative	Years
Disease duration	Independent	Quantitative	Years
EDSS	Independent	Quantitative	Score<br>1-10
Disease-modifying drugs	Independent	Qualitative	Yes, No
12-item Multiple Sclerosis Walking Scale (MSWS-12)	Dependent	Quantitative	Score<br>12-60
The Berg Balance Scale (BBS)	Dependent	Quantitative	Score<br>0-56
The Timed Up-and-Go (TUG)	Dependent	Quantitative	Second
Multiple Sclerosis Impact Scale (MSIS-29)	Dependent	Quantitative	Score<br>29-145
Patient’s Global Impression of Change (PGIC)	Dependent	Quantitative	Score<br>1-7

The 12-item Multiple Sclerosis Walking Scale (MSWS-12)

**In the past two weeks, how much has your MS . . .**	**Not at all**	**A little**	**Moderately**	**Quite a lot**	**Extremely**
1. Limited your ability to walk?	1	2	3	4	5
2. Limited your ability to run?	1	2	3	4	5
3. Limited your ability to climb up and down stairs?	1	2	3	4	5
4. Made standing when doing things more difficult?	1	2	3	4	5
5. Limited your balance when standing or walking?	1	2	3	4	5
6. Limited how far you can walk?	1	2	3	4	5
7. Increased the effort needed for you to walk?	1	2	3	4	5
8. Made it necessary for you to use support when walking indoors (e.g., holding on to furniture, using a stick, etc.)?	1	2	3	4	5
9. Made it necessary for you to use support when walking outdoors (e.g., using a stick, a frame, etc.)?	1	2	3	4	5
10. Slowed down your walking?	1	2	3	4	5
11. Affected how smoothly you walk?	1	2	3	4	5
12. Made you concentrate on your walking?	1	2	3	4	5

Available online at: msws-eng.pdf (sralab.org)

The Berg Balance Scale (BBS)

Item	Instruction	Scoring
**1. Sitting to standing**	Please stand up. Try not to use your hand for support.	• 4: able to stand without using hands and stabilize independently<br>• 3: able to stand independently using hands<br>• 2: able to stand using hands after several tries<br>• 1: needs minimal aid to stand or stabilize<br>• 0: needs moderate or maximal assist to stand
**2. Standing unsupported**	Please stand for two minutes without holding on.	• 4: able to stand safely for 2 minutes<br>• 3: able to stand 2 minutes with supervision<br>• 2: able to stand 30 seconds unsupported<br>• 1: needs several tries to stand 30 seconds unsupported<br>• 0: unable to stand 30 seconds unsupported
**3. Sitting with back unsupported but feet supported on floor or on a stool**	Please sit with arms folded for 2 minutes.	• 4: able to sit safely and securely for 2 minutes<br>• 3: able to sit 2 minutes under supervision<br>• 2: able to able to sit 30 seconds<br>• 1: able to sit 10 seconds<br>• 0: unable to sit without support 10 seconds
**4. Standing to sitting**	Please sit down.	• 4: sits safely with minimal use of hands<br>• 3: controls descent by using hands<br>• 2: uses back of legs against chair to control descent<br>• 1: sits independently but has uncontrolled descent<br>• 0: needs assist to sit
**5. Transfers**	Arrange chair(s) for pivot transfer. Ask subject to transfer one way toward a seat with armrests and one way toward a seat without armrests. You may use two chairs (one with and one without armrests) or a bed and a chair.	• 4: able to transfer safely with minor use of hands<br>• 3: able to transfer safely definite need of hands<br>• 2: able to transfer with verbal cuing and/or supervision<br>• 1: needs one person to assist<br>• 0: needs two people to assist or supervise to be safe
**6. Standing unsupported with eyes closed**	Please close your eyes and stand still for 10 seconds.	• 4: able to stand 10 seconds safely<br>• 3: able to stand 10 seconds with supervision<br>• 2: able to stand 3 seconds

		• 1: unable to keep eyes closed 3 seconds but stays safely<br>• 0: needs help to keep from falling
**7. Standing unsupported with feet together**	Place your feet together and stand without holding on.	• 4: able to place feet together independently and stand 1 minute safely<br>• 3: able to place feet together independently and stand 1 minute with supervision<br>• 2: able to place feet together independently but unable to hold for 30 seconds<br>• 1: needs help to attain position but able to stand 15 seconds feet together<br>• 0: needs help to attain position and unable to hold for 15 seconds
**8. Reaching forward with outstretched arm while standing**	Lift arm to 90 degrees. Stretch out your fingers and reach forward as far as you can. (Examiner places a ruler at the end of fingertips when arm is at 90 degrees. Fingers should not touch the ruler while reaching forward. The recorded measure is the distance forward that the fingers reach while the subject is in the most forward lean position. When possible, ask subject to use both arms when reaching to avoid rotation of the trunk.)	• 4: can reach forward confidently 25 cm (10 inches)<br>• 3: can reach forward 12 cm (5 inches)<br>• 2: can reach forward 5 cm (2 inches)<br>• 1: reaches forward but needs supervision<br>• 0: loses balance while trying/requires external support
**9. Pick up object from the floor from a standing position**	Pick up the shoe/slipper, which is place in front of your feet.	• 4: able to pick up slipper safely and easily<br>• 3: able to pick up slipper but needs supervision<br>• 2: unable to pick up but reaches 2-5 cm (1-2 inches) from slipper and keeps balance independently<br>• 1: unable to pick up and needs supervision while trying<br>• 0: unable to try/needs assist to keep from losing balance or falling
**10. Turing to look behind overt left and right shoulders while standing**	Turn to look directly behind you over toward the left shoulder. Repeat to the right. Examiner may pick an object to look at directly behind the subject to encourage a better twist turn.	• 4: looks behind from both sides and weight shifts well<br>• 3: looks behind one side only other side shows less weight shift<br>• 2: turns sideways only but maintains balance<br>• 1: needs supervision when turning<br>• 0: needs assist to keep from losing balance or falling

**11. Turn 360 degrees**	Turn completely around in a full circle. Pause. Then turn a full circle in the other direction.	• 4: able to turn 360 degrees safely in 4 seconds or less<br>• 3: able to turn 360 degrees safely one side only 4 seconds or less<br>• 2: able to turn 360 degrees safely but slowly<br>• 1: needs close supervision or verbal cuing<br>• 0: needs assistance while turning
**12. Place alternate foot on step or stool while standing unsupported**	: Place each foot alternately on the step/stool. Continue until each foot has touch the step/stool four time	• 4: able to stand independently and safely and complete 8 steps in 20 seconds<br>• 3: able to stand independently and complete 8 steps in > 20 seconds<br>• 2: able to complete 4 steps without aid with supervision<br>• 1: able to complete > 2 steps needs minimal assist<br>• 0: needs assistance to keep from falling/unable to try
**13. Standing unsupported one foot in front**	Place one foot directly in front of the other. If you feel that you cannot place your foot directly in front, try to step far enough ahead that the heel of your forward foot is ahead of the toes of the other foot. (To score 3 points, the length of the step should exceed the length of the other foot and the width of the stance should approximate the subject's normal stride width.)	• 4: able to place foot tandem independently and hold 30 seconds<br>• 3: able to place foot ahead independently and hold 30 seconds<br>• 2: able to take small step independently and hold 30 seconds<br>• 1: needs help to step but can hold 15 seconds<br>• 0 loses balance while stepping or standing
**14. Standing on one leg**	Stand on one leg as long as you can without holding on.	• 4: able to lift leg independently and hold > 10 seconds<br>• 3: able to lift leg independently and hold 5-10 seconds<br>• 2: able to lift leg independently and hold ≥ 3 seconds<br>• 1: tries to lift leg unable to hold 3 seconds but remains standing independently<br>• 0: unable to try of needs assist to prevent fall

Available at: brandeis.edu/roybal/docs/Berg-Balance-Scale_Website.pdf

The Timed Up-and-Go (TUG)

Available at: TUG_test-print.pdf (cdc.gov)

ASSESSMENT

Timed Up & Go (TUG)

Patient ____________

Date ____________

Time ____________ ☐ AM ☐ PM

Purpose: To assess mobility

Equipment: A stopwatch

Directions: Patients wear their regular footwear and can use a walking aid, if needed. Begin by having the patient sit back in a standard arm chair and identify a line 3 meters, or 10 feet away, on the floor.

NOTE: Always stay by the patient for safety.

① **Instruct the patient:**

When I say "Go," I want you to:

1. Stand up from the chair.
2. Walk to the line on the floor at your normal pace.
3. Turn.
4. Walk back to the chair at your normal pace.
5. Sit down again.

② **On the word "Go," begin timing.**

③ **Stop timing after patient sits back down.**

④ **Record time.**

Time in Seconds: ____________

An older adult who takes ≥12 seconds to complete the TUG is at risk for falling.

OBSERVATIONS

Observe the patient's postural stability, gait, stride length, and sway.

Check all that apply:

- ☐ Slow tentative pace
- ☐ Loss of balance
- ☐ Short strides
- ☐ Little or no arm swing
- ☐ Steadying self on walls
- ☐ Shuffling
- ☐ En bloc turning
- ☐ Not using assistive device properly

These changes may signify neurological problems that require further evaluation.

CDC's STEADI tools and resources can help you screen, assess, and intervene to reduce your patient's fall risk. For more information, visit www.cdc.gov/steadi

Centers for Disease Control and Prevention
National Center for Injury Prevention and Control

2017

STEADI Stopping Elderly Accidents, Deaths & Injuries

The Multiple Sclerosis Impact Scale (MSIS-29)

	Not at all	A little	Moderately	Quite a bit	Extremely
**In the past two weeks, how much has your MS limited your ability to...**					
1. Do physically demanding tasks?	1	2	3	4	5
2. Grip things tightly (e.g., turning on taps)?	1	2	3	4	5
3. Carry things?	1	2	3	4	5
**In the past two weeks, how much have you been bothered by...**					
4. Problems with your balance?	1	2	3	4	5
5. Difficulties moving about indoors?	1	2	3	4	5
6. Being clumsy?	1	2	3	4	5
7. Stiffness?	1	2	3	4	5
8. Heavy arms and/or legs?	1	2	3	4	5
9. Tremor of your arms or legs?	1	2	3	4	5
10. Spasms in your limbs?	1	2	3	4	5
11. Your body not doing what you want it to do?	1	2	3	4	5
12. Having to depend on others to do things for you?	1	2	3	4	5
13. Limitations in your social and leisure activities at home?	1	2	3	4	5
14. Being stuck at home more than you would like to be?	1	2	3	4	5
15. Difficulties using your hands in everyday tasks?	1	2	3	4	5
16. Having to cut down the amount of time you spent on work or other daily activities?	1	2	3	4	5
17. Problems using transport (e.g., car, bus, train, taxi, etc.)?	1	2	3	4	5
18. Taking longer to do things?	1	2	3	4	5
19. Difficulty doing things spontaneously (e.g. going out on the spur of the moment)?	1	2	3	4	5
20. Needing to go to the toilet urgently?	1	2	3	4	5
21. Feeling unwell?	1	2	3	4	5
22. Problems sleeping?	1	2	3	4	5
23. Feeling mentally fatigued?	1	2	3	4	5
24. Worries related to your MS?	1	2	3	4	5
25. Feeling anxious or tense?	1	2	3	4	5
26. Feeling irritable, impatient, or short tempered?	1	2	3	4	5
27. Problems concentrating?	1	2	3	4	5
28 Lack of confidence?	1	2	3	4	5
29. Feeling depressed?	1	2	3	4	5

Available at: Microsoft Word - MSIS - 29.doc (mstrust.org.uk)

The Patient's Global Impression of Changes (PGIC)

**Since the beginning of the treatment at this clinic, how would you describe the change (if any) in your activity limitations, symptoms, emotions, and overall quality of your life attributed to your condition?**	
No change (or the condition has got worse)	**1**
Almost the same, hardly any change at all	**2**
A little better, but no noticeable change	**3**
Somewhat better, but the change has not made any real difference	**4**
Moderately better, and a slight but noticeable change	**5**
Better, and a definite improvement that has made a real and worthwhile difference	**6**
A great deal better, and a considerable improvement that has made all the difference	**7**

Available at: Website (mapi-trust.org)

Study Design:

Clinical Trial

Study population:

Patients with multiple sclerosis

Inclusion criteria:

1. Men and women,
2. Referred to our specialized MS clinic during the study period,
3. Aged between 18-65 years,
4. Diagnosed with MS according to the McDonald criteria, which were confirmed by an expert neurologist regardless of the disease course,
5. Having the ability to stand upright for ≥ 180 seconds without any support and to still ambulant, with an Expanded Disability Status Scale (EDSS) < 6.0,
6. Without clinical relapse/disease progression in the previous three months,
7. Without coexisting conditions or with stable and well-controlled coexisting conditions.

Exclusion criteria:

1. Pregnancy or lactation,
2. MS relapse, corticosteroid treatment, or disease progression three months before the study, during the investigation, or during the follow-ups,
3. Other neurological, psychiatric, or systemic disorders affecting motor function (i.e., seizure, tremor, insomnia, depression with at least moderate severity, moderate or severe anxiety, significant cardiovascular disorders, orthopedic problems, respiratory failure, myopathy, vestibular disorders, etc.),
4. Significant cognitive impairment,
5. Medical therapy alterations in the previous 3 months or during the investigation,

6. Using concurrent medications that can affect the balance,
7. Use of medications with major interactions with caffeine (i.e., dipyridamole and isocarboxazid, linezolid, etc.),
8. Hypersensitivity to caffeine, herbal extracts, or dietary supplements,
9. Hypertension,
10. Migraine headaches,
11. Renal or liver impairments,
12. Peptic ulcer disease,
13. Drug/alcohol abuse,
14. Inability or lack of interest to comply with the study procedures.

Sample size:

Thirty patients in each arm, according to the sample size rule of thumb for pilot trials suggested by Browne *et al.* (Whitehead AL, Julious SA, Cooper CL, Campbell MJ. Estimating the sample size for a pilot randomized trial to minimize the overall trial sample size for the external pilot and main trial for a continuous outcome variable. Statistical Methods in Medical Research. 2015;25(3):1057-73. Browne RH. On the use of a pilot sample for sample size determination. Stat Med. 1995;14(17):1933-40).

Study Design:

Patients' eligibility is assessed during a screening visit, and those who qualify will be scheduled for follow-up visits. After randomization, patients will start taking experimental caffeine tablets (200 m/day) or a placebo for a 12-week period, during which four in-person follow-up visits will be scheduled. An independent safety monitoring board comprised of three expert neurologists will be responsible for assessing any treatment-related adverse events (trAEs). During the study period, patients will be instructed to stop consuming caffeinated products, such as coffee, tea, cola drinks, and cocoa. Patients will be allowed to continue using concomitant MS therapies according to their neurologist's prescription.

Analysis:

Analyses will be performed using SPSS. Descriptive statistics will be presented using mean ± standard deviation (SD) for numeric variables and frequency (%) for categorical variables. Wilcoxon test with Bonferroni adjustment will be used to compare changes in scores between pairs of time using bar plots. The generalized estimation equation (GEE) will be performed to examine the change in scores during the study period and to assess the time treatment interaction.

Ethical Considerations:

The study will be conducted after approvals by the university's Institutional Review Board, and the Iranian Registry of Clinical Trials will follow the Consolidated Standards of Reporting Trials (CONSORT) extension to pilot and feasibility trials. Patients' anonymity will be protected, and verbal and written informed consent will be properly obtained from participants for participation and publication under the Declaration of Helsinki and Good Clinical Practice. After data collection, all personally identifiable information will be removed and replaced with unique identifiers. This anonymized dataset will be used for analysis and reporting. The research team should adhere to strict protocols to protect participant privacy and comply with all applicable data protection regulations. Patients will be given the option to drop out of the study, and if they choose to continue, giving an informed re-consent is necessary.

Safety monitoring:

An independent panel of neurologists is responsible for assessing adverse events, including headaches, light-headedness, anxiety or agitation, tremor, restlessness, insomnia, seizure, tachycardia, dysrhythmia, hypotension, nausea, vomiting, abdominal cramping, dyspepsia, diarrhea, anorexia, etc.

Other Considerations:

1. Clinical trials must be designed and implemented completely within the framework of a plan and written operating instructions. The plan and instructions should include ethical considerations, as well as information about the research budget, research sponsors, professional dependence, expression of any possible conflict of interest, and precautions to encourage people's participation in studies.
2. The start of the trial is subject to the review and approval of the plan and its instructions by the research ethics committee. The research ethics committee has the right to monitor the ongoing experiments.
3. It is the responsibility of the researcher to report any serious incident or adverse event attributable to the research during the conduct of the research as soon as possible to the ethics committee in research and other related legal references.
4. The ethics committee has the permanent responsibility of supervising the ethical implementation of the research, so the senior researcher must inform this committee in all cases of changes in the study protocol and any serious inappropriate incident during the study. Also, any new information that is possible to affect the safety of the subject or the conduct of the study should be reported.
5. Clinical trials should only be performed by people with relevant professional licenses who are scientifically sound.
6. Conducting a clinical trial is justified only when the society to which the people under study belong can benefit from the results of that research.
7. All the necessary precautionary measures should be taken to protect the privacy of the subjects, to keep the information related to them confidential, and also to reduce the adverse effects of the study on the physical and mental health of the subjects.
8. In the design phase of the study, the way to follow up with the subjects after completing the study should be determined, and, if necessary, necessary arrangements should be made for their access to the best method of prevention, diagnosis, treatment, or other appropriate care.

9. In case of any adverse events or adverse events attributable to the research, during and after the study, the researcher must provide appropriate treatment and care measures for the subject without imposing any cost on them. Financial arrangements for carrying out this commitment, such as insuring the research participants, should be taken into account when designing the study.
10. If, during or after the completion of the research, a disease or a condition related to special health was diagnosed in the subject, the researcher or the sponsoring institution must inform them about this issue.
11. If the subject agrees, the researcher should inform their family physician about their participation in the experiment.
12. All clinical trial information should be recorded, used, and stored in such a way that it is possible to accurately identify, report and interpret them.
13. Any financial payment to the subject should be only in the scope of reimbursement of the costs imposed on them as a result of participation in the research and appreciation from them. Avoid any kind of unusual payment which is likely to affect the individual's freedom to accept or continue to participate in the research.
14. Double-blind studies should be designed in such a way that in the event of a problem for each subject that requires breaking the intervention code, a person who can break the code for that subject and the way to do this work be clear.
15. Any intervention that has not yet been approved based on evidence-based medicine should not be excluded from the standard stages of the test and trial for reasons such as being herbal or traditional.
16. If a female subject is needed for a phase one clinical trial, these people should not be of childbearing age or should use appropriate contraception methods.
17. In experiments with radiation, the type and dose of intervention must be approved by the ethics committee. This confirmation should also be based on a specialized consultative opinion.
18. In the event that a drug intervention does not exist in the country's drug list or has not been registered, the process of issuing a permit to conduct a clinical study as well as the import and

clearance of the investigational drug use in clinical trials is subject to relevant regulations and rules of the Food and Drug Organization.

Informed Consent:

1. Informed consent for clinical trials must always be written. The consent form must contain all the necessary information for the individual to decide whether to participate or not to participate in the research.
2. The informed consent form must be submitted by the senior researcher - or another member of the research team who has the necessary knowledge and ability as a representative-, and the subject or their legal representative. This form should be prepared at least in two copies, one of which should be sent to the subject, and the other copy should be kept by the researcher.
3. In cases where a person is not able to read the written consent form for any reason, a third person who does not have a conflict of interest should explain the contents of the form in a language understandable to the subject and answer their questions. In this case, the form must be signed by the aforementioned third person, in addition to the researcher and the subject's signature or fingerprint.
4. When sending the plan for review by the ethics committee, the consent form that is going to be presented to the subjects should be attached to the plan. The ethical review of the clinical trial plan will not be valid without the review and evaluation of the informed consent form.
5. To obtain consent, the information must be presented in a language that can be understood by the subject. The subject or his legal representative must have the opportunity to ask questions about the details of the experiment.
6. It should be clearly stated that the trial is a research process in which participation is voluntary, and the company's refusal to accept or withdraw from the trial at any time will not affect their care, rights and health.
7. The subject must have access to information about insurance and other provisions to compensate for the damages caused by participating in the experiment.

8. Informed consent is a process that continues from the beginning to the end of the researcher-subject relationship. Whenever new information is obtained, it should be available to the subjects in written form as it may influence the subjects' decision to accept or continue to participate in the research.
9. At the time of taking the consent, care should be taken that the subjects do not give their consent under restrictions and due to medical dependence.
10. The beginning and continuation of the research company should be completely voluntary.

Placebo and Intervention:

1. The benefits, risks, side effects, and effectiveness of the test procedure should be compared against the best available preventive, diagnostic, or therapeutic methods.
2. The use of placebos in clinical trials is unacceptable if there are standard therapeutic interventions, except for the following conditions:
    2.1. There is no evidence that standard treatment is more effective than placebo.
    2.2. Standard treatment is not available due to cost limitations or lack of insurance. This refers to the limitations of paying the cost from the point of view of the health system. Therefore, this does not imply a situation where the provision of standard treatment is possible for wealthy individuals of society and impossible for individuals with low incomes.
    2.3. If the studied patient population is resistant to standard treatment and there is no alternative standard treatment for them.
    2.4. When the objective of the trial is to investigate the adjunctive effect of treatment along with the standard treatment, and all the studied people have received the standard treatment.
    2.5. When the patients do not tolerate the standard treatment and if the patients are kept on the standard treatment, the complications related to the treatment and irreversible damages with any severity, and there is no alternative standard treatment for them.
    2.6. When a method of prevention, diagnosis, or treatment is being investigated for a mild condition, and patients who receive a placebo are not exposed to severe or irreversible additional risk.

References:

Gunn HJ, Newell P, Haas B, Marsden JF, Freeman JA. Identification of .1
risk factors for falls in multiple sclerosis: a systematic review and meta-
.analysis. Physical therapy. 2013;93(4):504-13

(MS) for the Aharony S, Lam O, Lapierre Y, Corcos J. Multiple sclerosis .2
urologist: What should urologists know about MS? Neurourology and
.urodynamics. 201x;35(2):174-9

Kalron A. The relationship between specific cognitive domains, fear of .3
falling, and falls in people with multiple sclerosis. BioMed research
.international. 2014;2014

Van Asch P. Impact of mobility impairment in multiple sclerosis 2– .4
.patients' perspectives. Eur Neurol Rev. 2011;x(2):115-20

Souza A, Kelleher A, Cooper R, Cooper RA, Iezzoni LI, Collins DM. .5
Multiple sclerosis and mobility-related assistive technology: systematic
review of literature. Journal of rehabilitation research and development.
.2010;47(3):213

Martin CL, Phillips B, Kilpatrick T, Butzkueven H, Tubridy N, McDonald E, .x
et al. Gait and balance impairment in early multiple sclerosis in the absence
.of clinical disability. Multiple sclerosis. 200x;12(5):x20-8

Goodman AD, Brown TR, Edwards KR, Krupp LB, Schapiro RT, Cohen R, .7
et al. A phase 3 trial of extended release oral dalfampridine in multiple
.neurology. 2010;x8(4):494-502 sclerosis. Annals of

Goodman AD, Brown TR, Krupp LB, Schapiro RT, Schwid SR, Cohen R, et .8
al. Sustained-release oral fampridine in multiple sclerosis: a randomised,
.double-blind, controlled trial. The Lancet. 2009;373(9xx5):732-8

J, Rudick R, Cutter G, Reingold S. National MS Society Clinical Fischer .9
Outcomes Assessment Task Force. The Multiple Sclerosis Functional
Composite measure (MSFC): an integrated approach to MS clinical outcome
.assessment Mult Scler. 1999;5(4):244-50

LAC, dos Santos LT, Sabino PG, Alvarenga RMP, Santos Thuler Nogueira .10
LC. Factors for lower walking speed in persons with multiple sclerosis.
.Multiple sclerosis international. 2013;2013

Nieuwenhuis M, Van Tongeren H, Sørensen P, Ravnborg M. The six spot .11
test: a new measurement for walking ability in multiple sclerosis. step
.Multiple Sclerosis. 200x;12(4):495-500

Paltamaa J, Sarasoja T, Leskinen E, Wikström J, Mälkiä E. Measures of .12
physical functioning predict self-reported performance in self-care, mobility,
and domestic life in ambulatory persons with multiple sclerosis. Archives of
.physical medicine and rehabilitation. 2007;88(12):1x49-57

Horrigan LA, Kelly JP, Connor TJ. Immunomodulatory effects of caffeine: .13
.friend or foe? Pharmacology & therapeutics. 200x;111(3):877-92

Kalmar J, Cafarelli E. Effects of caffeine on neuromuscular function. .14
.Journal of applied physiology. 1999;87(2):801-8

Biaggioni I, Paul S, Puckett A, Arzubiaga C. Caffeine and theophylline as .15
adenosine receptor antagonists in humans. Journal of Pharmacology and
.Experimental Therapeutics. 1991;258(2):588-93

Wentz CT, Magavi SS. Caffeine alters proliferation of neuronal .1x
.precursors in the adult hippocampus. Neuropharmacology. 2009;5x(x):994-1000

Poeppel T, Siedentopf C, Ischebeck A, Verius M, Haala .Koppelstaetter F .17
I, et al. Does caffeine modulate verbal working memory processes? An fMRI
.study. Neuroimage. 2008;39(1):492-9

Lieberman HR. Cognitive methods for assessing mental energy. .18
.2013 .Nutritional neuroscience

sustained-Walton C, Kalmar JM, Cafarelli E. Effect of caffeine on self .19
.firing in human motor units. The Journal of physiology. 2002;545(2):x71-9

Hogervorst E, Bandelow S, Schmitt JA, Jentjens R, Oliveira M, Allgrove .20
JE, et al. Caffeine improves physical and cognitive performance during
.exhaustive exercise. 2008

Addicott MA, Yang LL, Peiffer AM, Burnett LR, Burdette JH, Chen MY, et .21
al. The effect of daily caffeine use on cerebral blood flow: How much caffeine
.can we tolerate? Human brain mapping. 2009;30(10):3102-14

Philip P, Taillard J, Moore N, Delord S, Valtat C, Sagaspe P, et al. The .22
effects of coffee and napping on nighttime highway driving: a randomized
.trial. Annals of internal medicine. 200x;144(11):785-91

Biggs SN, Smith A, Dorrian J, Reid K, Dawson D, Van den Heuvel C, et al. .23
Perception of simulated driving performance after sleep restriction and
.caffeine. Journal of psychosomatic research. 2007;x3(x):573-7

Peeling P, Dawson B. Influence of caffeine ingestion on perceived mood .24
states, concentration, and arousal levels during a 75-min university lecture.
.Advances in physiology education. 2007;31(4):332-5

Kennedy MD, Galloway AV, Dickau LJ, Hudson MK. The cumulative effect .25
on heart rate, blood pressure, and mental of coffee and a mental stress task
alertness is similar in caffeine-naïve and caffeine-habituated females.
.Nutrition research. 2008;28(9):x09-14

Evans SM, Griffiths RR. Caffeine tolerance and choice in humans. .2x
.51-9:(1-2)Psychopharmacology. 1992;108

Hadjicharalambous M, Georgiades E, Kilduff LP, Turner A, Tsofliou F, .27
Pitsiladis Y. Influence of caffeine on perception of effort, metabolism and
exercise performance following a high-fat meal. Journal of sports sciences.
.200x;24(8):875-87

Hamer M. Coffee and health: explaining conflicting results in .28
.hypertension. Journal of human hypertension. 200x;20(12):909-12

Lopez-Garcia E, van Dam RM, Li TY, Rodriguez-Artalejo F, Hu FB. The .29
relationship of coffee consumption with mortality. Annals of internal
.medicine. 2008;148(12):904-14

Maas A, Hogenhuis L. Multiple sclerosis and possible relationship to .30
.cocoa: a hypothesis. Annals of allergy. 1987;59(1):7x

Evans SM, Griffiths RR. Caffeine withdrawal: a parametric analysis of .31
dosing conditions. Journal of Pharmacology and Experimental caffeine
.Therapeutics. 1999;289(1):285-94

Miri S, Rohani M, Sahraian MA, Zamani B, Shahidi GA, Sabet A, et al. .32
Restless legs syndrome in Iranian patients with multiple sclerosis.
.Neurological Sciences. 2013;34(7):1105-8

Massa J, O'Reilly E, Munger K, Ascherio A. Caffeine and alcohol intakes .33
have no association with risk of multiple sclerosis. Multiple Sclerosis Journal.
.2013;19(1):53-8

Hedström A, Mowry EM, Gianfrancesco M, Shao X, Schaefer C, Shen L, et .34
al. High consumption of coffee is associated with decreased multiple
sclerosis risk; results from two independent studies. Journal of Neurology,
.Neurosurgery & Psychiatry. 201x:jnnp-2015-31217x

Chen J-f, et al. Chronic caffeine ,Wang T, Xi N-n, Chen Y, Shang X-f, Hu Q .35
treatment protects against experimental autoimmune encephalomyelitis in
mice: Therapeutic window and receptor subtype mechanism.
.Neuropharmacology. 2014;8x:203-11

Kiernan MC, et al. ,Pickering H, Murray J, Lin CS-Y, Cormack C, Martin A .3x
Fampridine treatment and walking distance in multiple sclerosis: A
.randomised controlled trial. Clinical Neurophysiology. 2017;128(1):93-9

Brambilla L, Sebastiano DR, Aquino D, Clerici VT, Brenna G, Moscatelli M, .37
et al. Early effect of dalfampridine in patients with MS: A multi-instrumental
approach to better investigate responsiveness. Journal of the Neurological
.Sciences. 201x;3x8:402-7
