# Supplementary material for: Potential efficacy of caffeine ingestion on balance and mobility in patients with multiple sclerosis: Preliminary evidence from a single-arm pilot clinical trial
Source: PLoS One. 2024 Feb 13;19(2):e0297235. doi: 10.1371/journal.pone.0297235 (PMC10863863; doi:10.1371/journal.pone.0297235)
Supplement: S4 File — (PDF) [file pone.0297235.s004.pdf]

پروتکل کارآزمایی بالینی
مرکز ثبت کارآزمایی بالینی ایران

۱۴۰۲/۰۴/۰۴

تعیین تاثیر مصرف کافئین بر توانایی راه رفتن و تعادل در بیماران مبتلا به مالتیپل اسکلروزیس

چکیده پروتکل

چکیده

هدف:تعیین تاثیر مصرف کافئین بر توانایی راه رفتن و تعادل در بیماران مبتلا به مالتیپل اسکلروزیس طراحی انجام مطالعه:تصادفی-دو سویه کور-با کنترل با دارونما- جمعیت مورد مطالعه:حجم نمونه 30 نفر از افراد دارای MSحاضر در بیمارستان علی بن ابیطالب و انجمن ام اس زاهدان معیار اصلی ورود به مطالعه: شروع بیماری بیش از 60 روز قبل از ورودریا؛ عدم وجود سابقه آلرژی به مواد کافئین دار معیار خروج:هرگونه واکنش آلرژیک به کافئین حجم مطالعه:30 نفر مبتلا به بیماری مالتیپل اسکلروزیس مداخله مورد مطالعه: مصرف کافئین و تاثیر آن بر راه رفتن و تعادل در بیماران مبتلا به مالتیپل اسکلروزیس زمان مداخله:سه ماه پیامد اولیه:پیشرفت در امتیاز آزمون های EDSS,TUG,MSWS-12,PGIC,BBS,MSIS-29

اطلاعات عمومی

نام اختصاری

MS

اطلاعات ثبت در مرکز

شماره ثبت کارآزمایی در مرکز: **IRCT2017012332142N1**

تاریخ تایید ثبت در مرکز: **2017-03-27, ۱۳۹۶/۰۱/۰۷**

زمان‌بندی ثبت: **registered_while_recruiting**

آخرین بروز رسانی:

تعداد بروز رسانی‌ها: **0**

تاریخ تایید ثبت در مرکز

2017-03-27, ۱۳۹۶/۰۱/۰۷

اطلاعات تماس ثبت کننده

نام

افسون دادور

نام سازمان / نهاد

علوم پزشکی زاهدان

کشور

جمهوری اسلامی ایران

تلفن

+98 901 743 2529

آدرس ایمیل

afsoon.d@zaums.ac.ir

وضعیت بیمار گیری

بیمار گیری تمام شده

منبع مالی

هزینه مطالعه را معاونت پژوهشی پرداخت می نماید

تاریخ شروع بیمار گیری مورد انتظار

2017-03-10, ۱۳۹۵/۱۲/۲۰

تاریخ پایان بیمار گیری مورد انتظار

2017-05-05, ۱۳۹۶/۰۲/۱۵

تاریخ شروع بیمارگیری تحقق یافته

خالی

تاریخ پایان بیمارگیری تحقق یافته

خالی

تاریخ خاتمه کارآزمایی

خالی

عنوان علمی کارآزمایی

تعیین تاثیر مصرف کافئین بر توانایی راه رفتن و تعادل در بیماران مبتلا به مالتیپل اسکلروزیس

عنوان عمومی کارآزمایی

تاثیر مصرف کافئین بر راه رفتن و تعادل در بیماران مبتلا به مالتیپل اسکلروزیس

هدف اصلی مطالعه

درمانی

شرایط عمده ورود و عدم ورود به مطالعه

شرایط ورود: سن در محدوده 20-55؛ عدم وجود سابقه بیماری مرتبط با مطالعه؛ عدم وجود سابقه آلرژی به مواد کافئین دار؛ وزن بالای 40 کیلوگرم؛ شروع تشدید علائم بیماری ام اس از 60 روز قبل از غربالگری شرایط خروج: در صورت مشاهده هرگونه واکنش نسبتا شدید به کافئین؛ در صورت مشاهده هرگونه پیشرفت در بیماری

سن

از سن **20 ساله** تا سن **55 ساله**

جنسیت

هر دو

فاز مطالعه

مصداق ندارد

گروه‌های کور شده در مطالعه

اطلاعات موجود نیست

حجم نمونه کل

حجم نمونه پیش‌بینی شده: **30**

تصادفی سازی (نظر محقق)
اختصاص تصادفی به گروه‌های مداخله و کنترل
توصیف نحوه تصادفی سازی
کور سازی (به نظر محقق)
دو سویه کور
توصیف نحوه کور سازی
دارو نما
دارد
اختصاص به گروه‌های مطالعه
موازی
سایر مشخصات طراحی مطالعه
با استفاده از بلوک های تصادفی تصادفی سازی صورت خواهد گرفت

کد ثبت در سایر مراکز ثبت بین‌المللی

خالی

تاییدیه کمیته‌های اخلاق

1

کمیته اخلاق
نام کمیته اخلاق
مجتمع اداری پردیس دانشگاه علوم پزشکی و خدمات بهداشتی درمانی زاهدان
آدرس خیابان
زاهدان-میدان دکتر حسابی-بلوار خلیج فارس-مجتمع اداری پردیسدانشگاه علوم پزشکی و حدمات بهداشتی درمانی زاهدان
شهر
زاهدان
کد پستی
98167-43463
تاریخ تایید
2016-12-18, ۱۳۹۵/۰۹/۲۸
کد کمیته اخلاق
8029/کد اخلاقی طرح مربوطه : IR.ZAUMS.REC.1395. 236

بیماری‌های (موضوعات) مورد مطالعه

1

شرح
مالتیپل اسکلروزیس
کد ICD-10
G35
توصیف کد ICD-10
Multiple sclerosis

متغیر پیامد اولیه

1

شرح متغیر پیامد
توانایی راه رفتن و تعادل
مقاطع زمانی اندازه‌گیری
قبل از مداخله، 2، 4، 8 و 12 هفته بعد از مداخله
نحوه اندازه‌گیری متغیر
براساس نتایج آزمون های Kurtzke Expanded Disability Status Scale (EDSS),Twelve Item MS Walking Scale(MSWS-12),Patients' Global Impression of Change(PGIC),The Timed Up and Go (TUG) Test, Berg Balance SCALE(BBS),Multiple Sclerosis Impact Scale(MSIS-29)

متغیر پیامد ثانویه

1

شرح متغیر پیامد
عوارض جانبی دارو
مقاطع زمانی اندازه‌گیری
در تمام دوران انجام کارآزمایی در صورت هرگونه ناراحتی از سوی بیمار
نحوه اندازه‌گیری متغیر
براساس معیارهای ثبت شده بی خطری و تحمل بیماران را براساس AEs (ملاحظه عوارض جانبی)و SAEs(ملاحظه عوارض جانبی خطرناک)و علائم حیاتی و الکتروکاردیوگرام

گروه‌های مداخله

1

شرح مداخله
ماده مصرفی:کافئین، ترکیب شیمیایی:کافئین خالص شده ،غلظت:100درصد، دوز مصرف:2.5 میلی گرم به ازای هرکیلوگرم وزن فرد به صورت روزانه(کمتریت دوز مصرفی)، تعداد دفعات مصرف:روزی یک مرتبه، مدت مصرف:3ماه
طبقه بندی
غیره

2

شرح مداخله
ماده مصرفی:اسیدفولیک در قالب همان کپسول هاییکه میخواهیم در آن کافئین بریزیم. ترکیب شیمیایی:اسیدفولیک خالص،غلظت:100درصد، دوز مصرف:یک میلی گرم، تعداد دفعات مصرف:روزی یک مرتبه، مدت مصرف:یک ماه
طبقه بندی
دارو نما

مراکز بیمار گیری

1

مرکز بیمار گیری
نام مرکز بیمار گیری
انجمن ام اس زاهدان
نام کامل فرد مسوول
آقای مقتدری
آدرس خیابان
زاهدان-خیابان حکیم
شهر
زاهدان

2

مرکز بیمار گیری
نام مرکز بیمار گیری
بیمارستان علی بن ابی طالب زاهدان
نام کامل فرد مسوول
دکتر حامد امیری فرد
آدرس خیابان
شهر
زاهدان

حمایت کنندگان / منابع مالی

1

حمایت کننده مالی

نام سازمان / نهاد

دانشگاه علوم پزشکی زاهدان

نام کامل فرد مسوول

دکترمحسن طاهری

آدرس خیابان

زاهدان-میدان دکتر حسابی-بلوار خلیج فارس-پردیس دانشگاه علوم پزشکی و خدمات بهداشت درمان زاهدان

شهر

زاهدان

ردیف بودجه

کد بودجه

آیا منبع مالی همان سازمان یا نهاد حمایت کننده مالی است؟

بلی

عنوان منبع مالی

دانشگاه علوم پزشکی زاهدان

درصد تامین مالی مطالعه توسط این منبع

100

بخش عمومی یا خصوصی

خالی

مبدا اعتبار از داخل یا خارج کشور

خالی

طبقه بندی منابع اعتبار خارجی

خالی

کشور مبدا

طبقه بندی موسسه تامین کننده اعتبار

خالی

فرد مسوول پاسخگویی عمومی کارآزمایی

اطلاعات تماس

نام سازمان / نهاد

دانشگاه علوم پزشکی زاهدان

نام کامل فرد مسوول

دکتر حامد امیری فرد

موقعیت شغلی

متخصص مغز و اعصاب-عضو هیئت علمی داشگاه علوم پزشکی زاهدان

سایر حوزه‌های کاری/تخصص‌ها

آدرس خیابان

میدان دکتر حسابی-بلوار خلیج فارس-داشگاه علوم پزشکی زاهدان

شهر

زاهدان

کد پستی

تلفن

+98 54 1341 6708

فکس

ایمیل

dr.amirifard@gmail.com

آدرس صفحه وب

فرد مسوول پاسخگویی علمی مطالعه

اطلاعات تماس

نام سازمان / نهاد

دانشگاه علوم پزشکی زاهدان

نام کامل فرد مسوول

دکتر حامد امیری فرد

موقعیت شغلی

متخصص مغز واعصاب

سایر حوزه‌های کاری/تخصص‌ها

آدرس خیابان

میدان دکتر حسابی-بلوار خلیج فارس-دانشگاه علوم پزشکی زاهدان

شهر

زاهدان

کد پستی

تلفن

+98 54 1341 6708

فکس

ایمیل

dr.amirifard@gmail.com

آدرس صفحه وب

فرد مسوول به‌روز رسانی اطلاعات

اطلاعات تماس

نام سازمان / نهاد

دانشگاه علوم پزشکی زاهدان

نام کامل فرد مسوول

افسون دادور

موقعیت شغلی

دانشجوی پزشکی

سایر حوزه‌های کاری/تخصص‌ها

آدرس خیابان

شهر

کد پستی

تلفن

00

فکس

ایمیل

afsoond97@yahoo.com

آدرس صفحه وب

برنامه انتشار

فایل داده شرکت کنندگان (IPD)

خالی

پروتکل مطالعه

خالی

نقشه آنالیز آماری

خالی

فرم رضایتنامه آگاهانه

خالی

گزارش مطالعه بالینی

خالی

کدهای استفاده شده در آنالیز

خالی

نظام دسته‌بندی داده (دیکشنری داده)

خالی
