# Supplementary material for: Potential efficacy of caffeine ingestion on balance and mobility in patients with multiple sclerosis: Preliminary evidence from a single-arm pilot clinical trial
Source: PLoS One. 2024 Feb 13;19(2):e0297235. doi: 10.1371/journal.pone.0297235 (PMC10863863; doi:10.1371/journal.pone.0297235)
Supplement: S5 File — (PDF) [file pone.0297235.s005.pdf]

Clinical Trial Protocol
Iranian Registry of Clinical Trials

25 Jun 2023

Determine the effect of caffeine on the ability to walk and balance in patients with multiple sclerosis

Protocol summary

Summary

Objective: To determine the effect of caffeine on the ability to walk and balance in patients with multiple sclerosis study design: double-blind, randomized, placebo-controlled study population with: a sample of 30 subjects with MS now at Ali ibn Abi Talib and the MS Society of Zahedan main criteria for inclusion:the sickness getting started More than 60 days ago؛ the lack of a history of allergy to caffeine exclusion criteria: any allergic reaction to caffeine, the study: the study involved 30 patients with multiple sclerosis: the effects of caffeine on gait and balance in patients with multiple sclerosis intervention time: three months, the primary outcome: progress in EDSS,TUG,MSWS-12,PGIC,BBS,MSIS-29 test score

General information

Acronym

MS

IRCT registration information

IRCT registration number: **IRCT2017012332142N1**
Registration date: **2017-03-27, 1396/01/07**
Registration timing: **registered_while_recruiting**

Last update:
Update count: **0**

Registration date

2017-03-27, 1396/01/07

Registrant information

Name

afsoon dadvar

Name of organization / entity

medical university of zahedan

Country

Iran (Islamic Republic of)

Phone

+98 901 743 2529

Email address

afsoon.d@zaums.ac.ir

Recruitment status

Recruitment complete

Funding source

Vice chancellor for research,zahedan University of Medical Sciences

Expected recruitment start date

2017-03-10, 1395/12/20

Expected recruitment end date

2017-05-05, 1396/02/15

Actual recruitment start date

empty

Actual recruitment end date

empty

Trial completion date

empty

Scientific title

Determine the effect of caffeine on the ability to walk and balance in patients with multiple sclerosis

Public title

The effects of caffeine on walking and balance in patients with multiple sclerosis

Purpose

Treatment

Inclusion/Exclusion criteria

Inclusion criteria: age-related diseases in the range of 20-55; has no history of patients without a history of allergy to caffeine; aggravates symptoms of MS patients weighing more than 40 kg; beginning 60 days prior to screening for exclusion criteria: If there is any a severe reaction to caffeine; if you see any progress in disease

Age

From **20 years** old to **55 years** old

Gender

Both

Phase
N/A
Groups that have been masked
No information
Sample size
Target sample size: **30**
Randomization (investigator's opinion)
Randomized
Randomization description
Blinding (investigator's opinion)
Double blinded
Blinding description
Placebo
Used
Assignment
Parallel
Other design features
Using random blocks will be randomized

Secondary Ids

empty

Ethics committees

1

Ethics committee
Name of ethics committee
Ethics committee of zahedan University of Medical Sciences
Street address
hesabi square-khalige fars bulvar- medical univercity of zahedan
City
zahedan
Postal code
98167-43463
Approval date
2016-12-18, 1395/09/28
Ethics committee reference number
8029/کد اخلاقی طرح مربوطه : IR.ZAUMS.REC.1395. 236

Health conditions studied

1

Description of health condition studied
multiple sclerosis
ICD-10 code
G35
ICD-10 code description
Multiple sclerosis

Primary outcomes

1

Description
The ability to walk and balance
Timepoint
Before intervention, 2, 4, 8 and 12 weeks after intervention
Method of measurement
Based on the results of the Kurtzke Expanded Disability Status Scale (EDSS), Twelve Item MS Walking Scale (MSWS-12), Patients' Global Impression of Change (PGIC), The Timed Up and Go (TUG) Test, Berg Balance SCALE (BBS) , Multiple Sclerosis Impact Scale (MSIS-29)

Secondary outcomes

1

Description
Drug Side Effects
Timepoint
If any discomfort in the patient of all time trials
Method of measurement
Registered on the basis of safety and tolerability based on AEs (see side effects) and SAEs (serious adverse events observed) and vital signs and electrocardiogram

Intervention groups

1

Description
Consumption of caffeine, the chemical composition: pure caffeine concentration: 100%, dose: 2.5 mg per kilogram of body weight on a daily basis (minimum dose), the frequency of use: once a day, duration of use: 3 months
Category
Other

2

Description
Substance: folic acid in the form of capsules that you're going to throw Caffeine, the chemical composition: folic acid, concentration: 100%, dosage: One mg, the frequency of use: once a day, duration: one month
Category
Placebo

Recruitment centers

1

Recruitment center
Name of recruitment center
MS Society of Zahedan
Full name of responsible person
Street address
City
zahedan

2

Recruitment center
Name of recruitment center
Ali ibn Abi Talib Hospital in Zahedan
Full name of responsible person
Street address

City
zahedan

Sponsors / Funding sources

1

Sponsor
Name of organization / entity
zahedan University of Medical Sciences
Full name of responsible person
dr.mohsen tahery
Street address
Zahedan-square-hesabi doctor Persian Gulf Blvd. account-Medical Sciences and Health Services, Zahedan
City
zahedan
Grant name
Grant code / Reference number
Is the source of funding the same sponsor organization/entity?
Yes
Title of funding source
zahedan University of Medical Sciences
Proportion provided by this source
100
Public or private sector
empty
Domestic or foreign origin
empty
Category of foreign source of funding
empty
Country of origin
Type of organization providing the funding
empty

Person responsible for general inquiries

Contact
Name of organization / entity
zahedan University of Medical Sciences
Full name of responsible person
dr.hamed amirifard
Position
Neurologist-faculty at the zahedan University of Medical Sciences
Other areas of specialty/work
Street address
The doctor hesabi-Blvd. Persian Gulf-University of Medical Sciences
City
zahedan
Postal code
Phone
+98 54 1341 6708
Fax
Email
dr.amirifard@gmail.com
Web page address

Person responsible for scientific inquiries

Contact
Name of organization / entity
zahedan University of Medical Sciences
Full name of responsible person
dr amiri fard
Position
Neurologist
Other areas of specialty/work
Street address
The doctor hesabi-Blvd. Persian Gulf-University of Medical Sciences
City
zahedan
Postal code
Phone
+98 54 1341 6708
Fax
Email
dr.amirifard@gmail.com
Web page address

Person responsible for updating data

Contact
Name of organization / entity
zahedan University of Medical Sciences
Full name of responsible person
afsoon dadvar
Position
medical student
Other areas of specialty/work
Street address
City
Postal code
Phone
00
Fax
Email
afsoond97@yahoo.com
Web page address

Sharing plan

Deidentified Individual Participant Data Set (IPD)
empty
Study Protocol
empty
Statistical Analysis Plan
empty
Informed Consent Form
empty
Clinical Study Report
empty
Analytic Code
empty
Data Dictionary
empty
